# CRF_1_ Receptor Signaling via the ERK1/2-MAP and Akt Kinase Cascades: Roles of Src, EGF Receptor, and PI3-Kinase Mechanisms

**DOI:** 10.3389/fendo.2019.00869

**Published:** 2019-12-12

**Authors:** G. Karina Parra-Mercado, Alma M. Fuentes-Gonzalez, Judith Hernandez-Aranda, Monica Diaz-Coranguez, Frank M. Dautzenberg, Kevin J. Catt, Richard L. Hauger, J. Alberto Olivares-Reyes

**Affiliations:** ^1^Laboratory of Signal Transduction, Department of Biochemistry, Center for Research and Advanced Studies of the National Polytechnic Institute, CINVESTAV-IPN, Mexico City, Mexico; ^2^Novaliq GmbH, Heidelberg, Germany; ^3^Section on Hormonal Regulation, Program on Developmental Endocrinology and Genetics, National Institute of Child Health and Human Development, Bethesda, MD, United States; ^4^Center of Excellence for Stress and Mental Health, VA Healthcare System, San Diego, CA, United States; ^5^Department of Psychiatry, University of California, San Diego, San Diego, CA, United States

**Keywords:** corticotropin-releasing factor, CRF_1_ receptor, EGF receptor transactivation, ERK1/2, Src, PI3K/Akt

## Abstract

In the present study, we determined the cellular regulators of ERK1/2 and Akt signaling pathways in response to human CRF_1_ receptor (CRF_1_R) activation in transfected COS-7 cells. We found that Pertussis Toxin (PTX) treatment or sequestering Gβγ reduced CRF_1_R-mediated activation of ERK1/2, suggesting the involvement of a G_i_-linked cascade. Neither G_s_/PKA nor G_q_/PKC were associated with ERK1/2 activation. Besides, CRF induced EGF receptor (EGFR) phosphorylation at Tyr^1068^, and selective inhibition of EGFR kinase activity by AG1478 strongly inhibited the CRF_1_R-mediated phosphorylation of ERK1/2, indicating the participation of EGFR transactivation. Furthermore, CRF-induced ERK1/2 phosphorylation was not altered by pretreatment with batimastat, GM6001, or an HB-EGF antibody indicating that metalloproteinase processing of HB-EGF ligands is not required for the CRF-mediated EGFR transactivation. We also observed that CRF induced Src and PYK2 phosphorylation in a Gβγ-dependent manner. Additionally, using the specific Src kinase inhibitor PP2 and the dominant-negative-SrcYF-KM, it was revealed that CRF-stimulated ERK1/2 phosphorylation depends on Src activation. PP2 also blocked the effect of CRF on Src and EGFR (Tyr^845^) phosphorylation, further demonstrating the centrality of Src. We identified the formation of a protein complex consisting of CRF_1_R, Src, and EGFR facilitates EGFR transactivation and CRF_1_R-mediated signaling. CRF stimulated Akt phosphorylation, which was dependent on G_i_/βγ subunits, and Src activation, however, was only slightly dependent on EGFR transactivation. Moreover, PI3K inhibitors were able to inhibit not only the CRF-induced phosphorylation of Akt, as expected, but also ERK1/2 activation by CRF suggesting a PI3K dependency in the CRF_1_R ERK signaling. Finally, CRF-stimulated ERK1/2 activation was similar in the wild-type CRF_1_R and the phosphorylation-deficient CRF_1_R-Δ386 mutant, which has impaired agonist-dependent β-arrestin-2 recruitment; however, this situation may have resulted from the low β-arrestin expression in the COS-7 cells. When β-arrestin-2 was overexpressed in COS-7 cells, CRF-stimulated ERK1/2 phosphorylation was markedly upregulated. These findings indicate that on the base of a constitutive CRF_1_R/EGFR interaction, the G_i_/βγ subunits upstream activation of Src, PYK2, PI3K, and transactivation of the EGFR are required for CRF_1_R signaling via the ERK1/2-MAP kinase pathway. In contrast, Akt activation via CRF_1_R is mediated by the Src/PI3K pathway with little contribution of EGFR transactivation.

## Introduction

Behavioral, cognitive, neuroendocrine, and autonomic responses to stress are regulated by CRF_1_ and CRF_2_ receptors (CRF_1_R and CRF_2_R) (1–3). The preferred mode of signal transduction by both CRF receptors was initially believed to be activation of the G_s_/adenylyl cyclase/PKA signaling pathway (1–3). Subsequently, CRF_1_R and CRF_2_R were also found to signal *via* the PLC/PKC cascade stimulating intracellular calcium mobilization and IP3 formation (1–4). Besides, both CRF receptors can activate mitogen-activated protein (MAP) kinase cascades in neuronal, cardiac, and myometrial cells endogenously expressing CRF_1_R or CRF_2_R and in recombinant cell lines expressing either receptor (2, 3, 5, 6). Several reports suggested that cellular background directed CRF_1_R to signal selectively via a specific MAP kinase pathway. For example, agonist-activated CRF_1_Rs stimulated phosphorylation of ERK1/2 and p38 MAP kinases in PC12 and fetal microglial cells (7, 8) while CRF_1_Rs activated ERK1/2 but not JNK and p38 in CHO cells (9). In human mast cells and HaCaT keratinocytes, on the other hand, CRF_1_Rs induce phosphorylation of p38 but not ERK or JNK MAP kinases (10, 11). Most studies suggest, however, that the ERK1/2 cascade is the MAP kinase pathway preferentially used by CRF receptors (5, 9, 12, 13).

Signaling via the cyclic AMP (cAMP)-PKA pathway by G_s_-coupled GPCRs has been proposed to mediate upstream activation of the ERK cascade in cells with high B-Raf expression (14). Consistent with this concept, PKA regulates CRF_1_R-mediated ERK activation and ERK-dependent Elk1 transcription in AtT-20 pituitary cells that express high B-Raf levels (15). Kageyama et al. (16) found, however, that ERK activation by CRF_1_R was mediated by a PKA-independent mechanism in AtT-20 cells. Moreover, other studies have reported that PKA does not play a role in CRF_1_R ERK signaling in rat CATH.a and rat fetal microglial cells, locus coeruleus neurons, and transfected CHO cells (8, 9, 12, 17). CRF_1_R can also activate the ERK1/2 cascade via a PKC-dependent mechanism, based on data showing that pretreatment with a PLC or PKC inhibitor blocked urocortin 1 (Ucn1)-stimulated phosphorylation of ERK1/2 in CRF_1_R-expressing human myometrial, CHO, and HEK293 cells (12, 13), and in rat hippocampal neurons (18). PKC inhibitor pretreatment, however, failed to block CRF- and Ucn1-stimulated ERK1/2 phosphorylation in CRF_1_R-expressing pituitary AtT20 cells and brain-derived CATH.a cells expressing both CRF receptors (12, 16). These findings suggest that cellular background may also govern the ability of PKA or PKC pathways to regulate CRF_1_R ERK1/2 signaling similar to its possible role in mediating CRF_1_R selective activation of a specific MAP kinase cascade.

MEK1/2-mediated phosphorylation of ERK1/2 at Thr^202^ and Tyr^204^ during CRF_1_R and CRF_2_R signaling in various cell lines has been confirmed by inhibiting ERK1/2 activation with PD98059 (2, 9, 12, 13, 19). Inhibiting C-Raf function by pretreatment with R1-K1 inhibitor or blocking Ras activation by transfection with the dominant-negative mutant RasS17N inhibited Ucn1-stimulated ERK1/2 phosphorylation in CRF_1_R-expressing CHO and HEK293 cells (5, 12). CRF_2_R activation by urocortin 2 (Ucn2) and urocortin 3 (Ucn3) has also been shown to signal via the Ras→C-Raf→MEK1/2 cascade in rat cardiomyocytes, based on the ability of manumycin A (a Ras inhibitor) and R1-K1 to abolish ERK1/2 phosphorylation (19). Other research has provided evidence for a phosphoinositide 3-kinase (PI3K)-dependent mechanism contributing to CRF_1_R- and CRF_2_R-mediated ERK1/2 activation in HEK293, CHO, A7r5, and CATH.a cells (5, 9, 12). EGF receptor (EGFR) transactivation, possibly by matrix metalloproteinase (MMP)-mediated ligand release, has been shown to contribute to Ucn1-stimulated ERK1/2 phosphorylation in HEK293 cells, although the mechanisms for CRF_1_R-mediated transactivation of the EGFR were not determined (5). Furthermore, another study reported that CRF receptor ERK signaling in the mouse atrial HL-1 cardiomyocyte line involved activation of Src (20).

In addition, activation of CRF_1_R or CRF_2(b)_R can stimulate phosphorylation of Akt (5, 21). CRF_2(b)_R Akt signaling in HEK293 cells is mediated by pertussis-sensitive G proteins and PI3K but not by cAMP-stimulated activation of PKA or EPAC, or by PKC (21). The mechanisms regulating Akt signal transduction by CRF_1_R, however, have not been investigated. Because upstream kinase pathway mediation of CRF_1_R signal transduction via the ERK and Akt cascades are not well-understood, the primary goal of this study was to test the hypothesis that Src tyrosine kinase and EGFR transactivation are essential regulators of these CRF_1_R signaling pathways. We also sought to determine the relative importance of G protein βγ subunits, second messenger kinases, and PI3K in the activation of the ERK1/2 and Akt cascades by the CRF_1_R. The results of our study indicate that upstream utilization of Src and PI3K are involved in ERK and Akt signal transduction by the agonist-activated CRF_1_R in COS-7 cells, without mediation by PKA and PKC, while transactivation of the EGFR is mainly required for CRF_1_R to stimulate phosphorylation of ERK but not for Akt activation.

## Materials and Methods

### Materials

General reagents utilized were as follows: (i) DMEM, fetal bovine serum (FBS), antibiotic solutions and other cell culture reagents from Invitrogen/GIBCO (Carlsbad, CA); (ii) Pertussis Toxin, reagents for electrophoresis and other highly pure chemicals from Sigma-Aldrich (St. Louis, MO). Human/rat CRF was purchased from Bachem (Torrance, CA). Phorbol 12-myristate 13-acetate (PMA), and the following specific inhibitors were purchased from Calbiochem (La Jolla, CA): Src inhibitor PP2; EGFR tyrosine kinase inhibitor AG1478; PKA inhibitor H89; PKC inhibitor bisindolylmaleimide I, BIM; PI3K inhibitors wortmannin and LY294002; MMP inhibitor GM6001, and Protease Inhibitor Cocktail Set III. BB-94 (batimastat) was obtained from British Biotechnology Ltd (Oxford, UK). Antibodies for Western blots were obtained from the following sources: (i) phospho-p44/42 MAP kinase (Thr^202^/Tyr^204^), total ERK1/2, total EGFR and phospho-c-Src (Tyr^416^) from Cell Signaling Technology (Beverly, MA); (ii) total ERK2, total c-Src and total Akt from Santa Cruz Biotechnology (Santa Cruz, CA); (iii) phospho-EGFR (Tyr^1068^), phospho-EGFR (Tyr^1173^) and phospho-EGFR (Tyr^845^) from Biosource-Invitrogen (Carlsbad, CA); (iv) phospho-Akt (Ser^473^) from Biosource International (Camarillo, CA); (v) phospho-PYK2 (Tyr^402^) from Calbiochem (La Jolla, CA); (vi) polyclonal anti-human HB-EGF antibody from R&D Systems (Minneapolis, MN); (vii) secondary antibodies conjugated to horseradish peroxidase from Zymed Laboratories (San Francisco, CA).

### DNA Constructs

The HA-epitope tagging human CRF_1_R (HA-CRF_1_R), the HA-CRF_1_R-Δ386 mutant, and the β-arrestin-2 constructs were previously described (22–24). Plasmid pRK5 encoding the carboxyl terminus of βARK that contains its βγ-binding domain (ct-βARK) was kindly provided by Dr. W. Koch (Center for Translational Medicine, Temple University, Philadelphia, PA), which is a scavenger for G protein βγ subunits (25). The expression vector pCEFL-SrcYF-KM, which contains the inactive form of SrcYF, SrcYF-KM (dominant-negative, dn-Src), was kindly provided by Dr. Silvio Gutkind (Department of Pharmacology, UCSD, La Jolla, CA) (26). Plasmid pUSEamp encoding dominant-negative Akt-K179M (dn-Akt) was from Upstate Biotechnology (Lake Placid, NY).

### Cell Culture and Transfection

COS-7 cells (from the American Type Culture Collection) were cultured at 37°C in a humidified atmosphere of 95% air, 5% CO_2_, in DMEM supplemented with 10% FBS, 100 μg/ml streptomycin, and 100 units/ml penicillin (COS-7 growth medium). Transient transfections were performed using LipofectAMINE (Life Technologies: Gaithersburg, MD) as described previously (27). Cells were seeded at 8 × 10^5^ cells/10-cm dish in COS-7 medium and cultured for 3 days before transfection. COS-7 cells were transfected in 5 ml/dish OptiMEM containing 10 μg/ml LipofectAMINE with empty vector, pcDNA3 encoding the HA-CRF_1_R or the HA-CRF_1_R-Δ386 mutant. In certain experiments, cells were co-transfected with plasmids containing: HA-CRF_1_R and mock (empty vector); HA-CRF_1_R and ct-βARK; HA-CRF_1_R and dn-Src; HA-CRF_1_R and dn-Akt, or HA-CRF_1_R and full-length β-arrestin-2. After replacing the transfection medium with fresh growth medium, transfected COS-7 cells were cultured for 1 day. Subsequently, cells were re-seeded in 6-well plates and cultured for an additional day prior to the experiment.

### Western Blot Methods

The protocols for measuring total and phosphorylated ERK1/2, c-Src, PYK2, Akt, and EGFR have been previously published (28, 29). After cells were cultured to 60–70% confluence, they were serum-deprived for 24 h. On the day of the experiment, cells were treated with the indicated ligands and inhibitors. No significant changes in the basal level of ERK1/2 or Akt phosphorylation were observed in cells pretreated with inhibitors, except for BIM, which showed a small increase in ERK1/2 activation (Supplementary Figure S1). After treatment, cells were placed on ice, the media was aspirated, and the cells were washed twice with ice-cold PBS and lysed in 100 μl of Laemmli sample buffer 1X. The lysates were briefly sonicated, heated at 95°C for 5 min, and centrifuged for 5 min at 14,000 rpm. Resulting supernatants were loaded in separate lanes of a 10% SDS-PAGE gels and electrophoresed. Next, Western transfer on to PDVF membranes was completed. The Western blots were then probed with specific antibodies targeting phosphorylated and non-phosphorylated forms of ERK1/2, c-Src, PYK2, Akt, and EGFR for primary immunodetection. After blots were probed with horseradish peroxidase-conjugated secondary antibody, protein bands were visualized with enhanced chemiluminescence ECL reagent (Amersham Pharmacia Biotech, Piscataway, NJ or Pierce Biotechnology, Rockford, IL) and scanned using the GS-800 Calibrated Imaging Densitometer (Bio-Rad). The labeled bands were quantified using the Quantity One 4.6.3 software program (Bio-Rad).

### Co-immunoprecipitation Assay

COS-7 cells transfected with HA-CRF_1_R were grown in 10-cm dishes and serum-deprived for 24 h before treatment with 100 nM CRF for 10 min at 37°C. Cells were washed twice with ice-cold PBS and lysed in Nonidet P-40 solubilization buffer (50 mM Tris-HCl, 150 mM NaCl, 2 mM Orthovanadate, 1 mM NaF, 1% Nonidet P-40, 10% Glycerol, 2 mM EDTA, pH 7.4, containing protease inhibitors). After immunoprecipitation of HA-CRF_1_Rs with anti-HA monoclonal antibody (HA.11; Covance, San Diego, CA) and protein A/G PLUS-Agarose (Santa Cruz Biotechnology, CA), the proteins were resolved by SDS-PAGE, Western blotted, and probed with anti-EGFR polyclonal or anti-HA monoclonal antibodies, followed by a horseradish peroxidase conjugate to identify co-immunoprecipitated proteins. Blots were also stripped with stripping buffer (100 nM Glycine-HCl, pH 2.7) and reprobed with anti-c-Src polyclonal antibody. Western blot detection of co-immunoprecipitated Src was carried out as described above. Blots were visualized and quantified, as indicated above.

### Statistical Analysis

Data are presented as mean ± S.E.M. Analyses of variances (ANOVAs) across experimental groups were performed using PRISM™, Version 8.0 for macOS (GraphPad Software, Inc., San Diego, CA). If the one-way ANOVA was statistically significant, planned *post-hoc* analyses were performed using Dunnet or Bonferroni's multiple comparison tests to determine individual group differences.

## Results

### CRF-Induced ERK1/2 Phosphorylation Is Dependent on G_i_ Protein and the Gβγ Subunits

**Figure 1:**
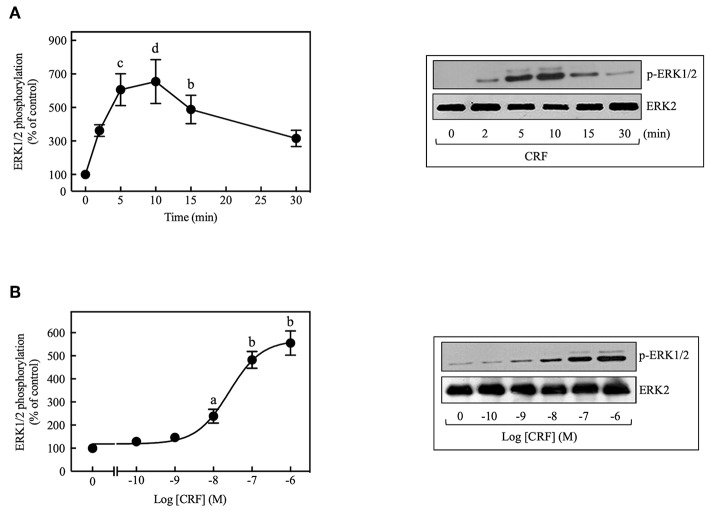
CRF induces ERK1/2 phosphorylation in COS-7 expressing CRF_1_Rs. **(A)** COS-7 cells expressing HA-CRF_1_Rs were treated with 100 nM CRF for the indicated times. **(B)** COS-7 cells expressing HA-CRF_1_Rs were exposed to the indicated concentration of CRF for 5 min. Total cell lysates were separated by SDS-PAGE and analyzed by immunoblotting with anti-p-ERK1/2 Thr^202^/*Tyr*^204^, as described in Materials and Methods. ERK1/2 phosphorylation was quantitated by densitometry, and mean values were plotted from three to five independent experiments. Vertical lines represent the S.E.M. Representative immunoblots are presented. Western blots were also probed for total ERK, showing equal loading. **(A)** ^b^*p* < 0.01, ^c^*p* < 0.001, ^d^*p* < 0.0001 vs. 0 min. **(B)** ^a^*p* < 0.05, ^b^*p* < 0.01 vs. 0 M.

CRF treatment (100 nM) of COS-7 cells transiently transfected with HA-CRF_1_R caused transient phosphorylation of ERK1/2 that reached a peak at 5–10 min and declined thereafter toward the basal level over the next 30 min (Figure 1A). CRF (100 nM) also caused time-dependent phosphorylation of ERK1/2 in CRF_1_R-expressing HEK293 and CHO-K1 cells (data not shown), but the rate and magnitude of CRF-induced ERK1/2 activation was considerably less in these cell lines compared to COS-7 cells. In contrast, CRF_1_Rs expressed in SK-N-MC neuroblastoma cells (4) failed to signal *via* the ERK1/2 cascade while fibroblast growth factor induced strong ERK1/2 phosphorylation in this cell line (Supplementary Figure S2). Therefore, all subsequent experiments studying ERK1/2 signaling were performed in COS-7 cells transfected with HA-CRF_1_R cDNA. CRF-induced ERK1/2 activation was concentration-dependent, with a significant increase at 10 nM CRF (~2.4-fold increase over control) and maximal effect over the 0.1–1 μM range (~5.2-fold increase over control, Figure 1B). The EC_50_ was 25 nM and the maximum occurred at 100 nM for the CRF-induced ERK1/2 phosphorylation.

**Figure 2:**
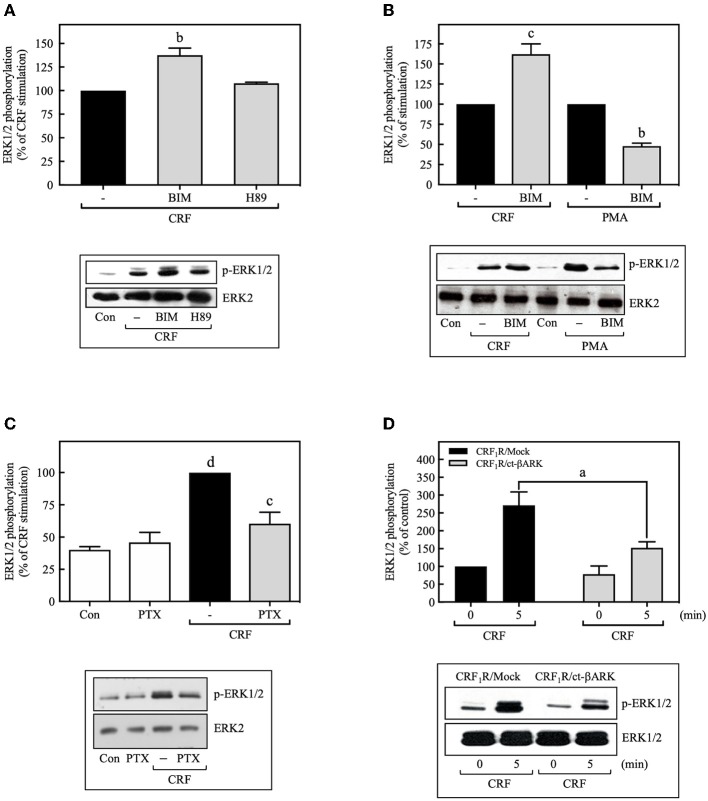
CRF-induced ERK1/2 phosphorylation is dependent on G_i_ protein. COS-7 cells expressing HA-CRF_1_Rs were pretreated with 0.5 μM H89 **(A)** or 1 μM BIM (bisindolylmaleimide I) **(A,B)** for 30 min, before stimulation with 100 nM CRF **(A,B)** for 5 min or 100 nM PMA **(B)** for 15 min. **(C)** COS-7 cells expressing HA-CRF_1_Rs were pretreated with 100 ng/ml PTX for 15 h, before stimulation with 100 nM CRF for 5 min. **(D)** COS-7 cells co-transfected with a plasmid pRK5 encoding the carboxyl terminus of βARK that contains its βγ-binding domain (ct-βARK) or an empty control vector (Mock) and the pcDNA3-HA-CRF_1_R expression vector were stimulated with 100 nM CRF for 5 min. Total cell lysates were separated by SDS-PAGE and analyzed by immunoblotting with anti-p-ERK1/2 Thr^202^/Tyr^204^, as described in Materials and Methods. ERK1/2 phosphorylation was quantitated by densitometry, and mean values were plotted from five independent experiments. Vertical lines represent the S.E.M. Western blots were also probed for total ERK showing equal loading. **(A)** ^b^*p* < 0.01 vs. CRF (–). **(B)** ^c^*p* < 0.001 vs. CRF (–); ^b^*p* < 0.01 vs. PMA (–). **(C)** ^d^*p* < 0.0001 vs. Con or PTX; ^c^*p* < 0.001 vs. CRF (–). **(D)** ^a^*p* < 0.05 vs. CRF_1_R/Mock (5 min).

Most of the known actions of CRF_1_Rs are mediated through the G_s_/PKA signaling cascade, but some of the physiological actions of CRF are also known to occur through activation of G_q_ or G_i_ proteins (30). To determine the contributions of G_s_/PKA-dependent mechanisms to MAP kinase activation, COS-7 cells were pretreated with the PKA inhibitor H89 (500 nM) for 30 min prior to stimulation with CRF (100 nM). As shown in Figure 2A, the PKA inhibitor failed to inhibit CRF-stimulated ERK1/2 phosphorylation. Furthermore, the magnitude of CRF_1_R-mediated activation of ERK1/2 was similar in COS-7 cells pretreated for 30 min with the highly selective PKA inhibitor Rp-cAMP (0–100 μM) or vehicle (Supplementary Figure S3). We next explored the involvement of G_q_/PKC in CRF_1_R ERK signaling. A 30-min pretreatment of COS-7 cells with the PKC inhibitor BIM (1 μM), increased (~1.6-fold increase over CRF stimulation) rather than decreased CRF-stimulated ERK1/2 activation (Figure 2B). In contrast, BIM pretreatment inhibited ERK1/2 phosphorylation resulting from PMA-induced PKC activation (Figure 2B). Thus, our data suggest that neither G_s_/PKA nor G_q_/PKC are required for the CRF_1_R-mediated ERK1/2 signaling in COS-7 cells.

On the other hand, the release of Gβγ subunits during GPCR coupling to G protein, particularly through G_i_, has an important role in downstream signaling in the ERK1/2 cascade (31, 32). Thus, we examined the role of G_i_ and Gβγ in CRF-stimulated ERK1/2 activation by two different experimental approaches: treatment with the G_i_ protein inhibitor pertussis toxin (PTX) and by co-transfecting COS-7 cells with plasmids encoding the carboxyl terminus of βARK containing its βγ-binding domain (ct-βARK) (Supplementary Figure S4), which sequesters βγ, and the CRF_1_R. COS-7 cells expressing CRF_1_Rs pretreated with 100 ng/ml PTX showed a marked reduction in CRF-induced ERK1/2 phosphorylation (Figure 2C), suggesting the coupling of CRF_1_R to G_i_ to mediate ERK activation. Moreover, overexpressing ct-βARK in COS-7 cells co-expressing CRF_1_Rs significantly reduced CRF-induced ERK1/2 phosphorylation by ~40% (Figure 2D). Thus, our data implicate Gβγ subunits from PTX-sensitive heterotrimeric G proteins in CRF_1_R-mediated activation of ERK1/2.

### Transactivation of the EGFR During CRF_1_R ERK1/2 Signaling

**Figure 3:**
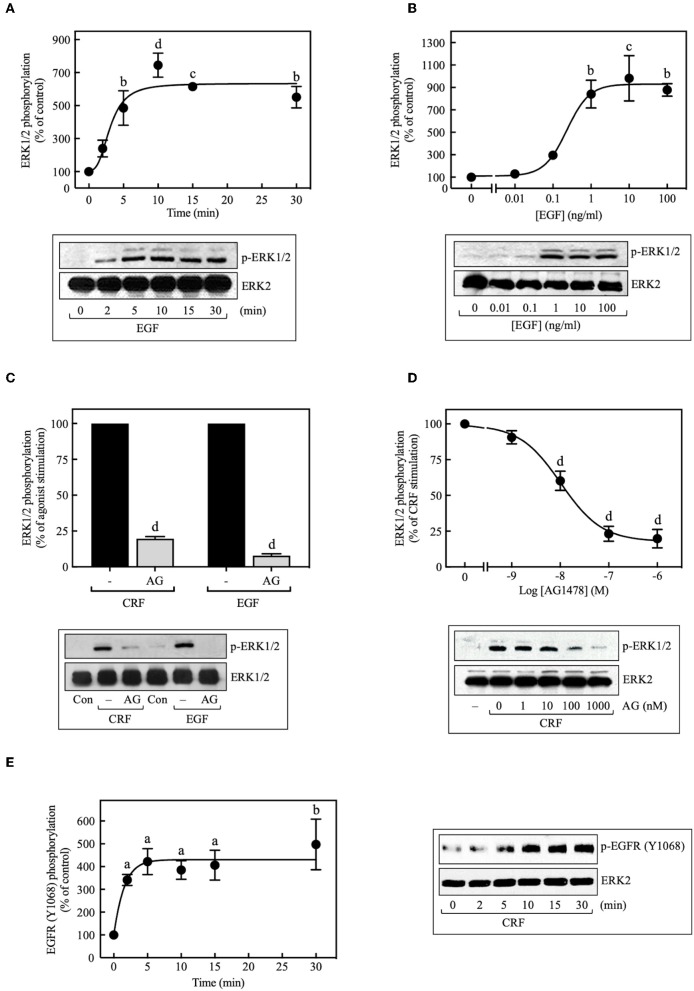
Involvement of EGFR transactivation in CRF-stimulated ERK1/2 phosphorylation. COS-7 cells expressing HA-CRF_1_Rs were treated with 10 ng/ml EGF for the indicated times **(A)** or exposed to the indicated concentration **(B)** of EGF for 10 min. COS-7 cells expressing HA-CRF_1_Rs were pretreated with 100 nM **(C)** or the indicated concentrations of AG1478 (AG) **(D)** for 30 min, before stimulation with 100 nM CRF for 5 min **(C,D)** or 10 ng/ml EGF for 10 min **(C)**. **(E)** Effect of 100 nM CRF on EGFR phosphorylation at Tyr^1068^. Total cell lysates were separated by SDS-PAGE and analyzed by immunoblotting with anti-p-ERK1/2 Thr^202^/Tyr^204^ **(A–D)** or anti-p-EGFR Tyr^1068^ **(E)**, as described in Materials and Methods. ERK1/2 and EGFR phosphorylation were quantitated by densitometry, and mean values were plotted from three to five independent experiments. Vertical lines represent the S.E.M. Western blots were also probed for total ERK, showing equal loading. **(A)** ^b^*p* < 0.01, ^c^*p* < 0.001, ^d^*p* < 0.0001 vs. 0 min. **(B)** ^b^*p* < 0.01, ^c^*p* < 0.001 vs. 0 ng/ml. **(C)** ^d^*p* < 0.0001 vs. CRF (–); ^d^*p* < 0.0001 vs. EGF (–). **(D)** ^d^*p* < 0.0001 vs. 0 M. **(E)** ^a^*p* < 0.05, ^b^*p* < 0.01 vs. 0 min.

Because transactivation of receptor tyrosine kinases (RTKs), especially the EGFR, is often an important mechanism used by GPCRs to activate ERK1/2 (33, 34), we investigated the role of EGFR transactivation in CRF_1_R-mediated ERK signaling. In COS-7 cells transiently transfected with HA-CRF_1_R, EGF stimulation of the endogenous EGFRs caused ERK1/2 phosphorylation in a time-dependent manner, reaching a maximum effect after 5 min of stimulation, which persisted for at least 30 min (~6.0-fold increase over time 0, Figure 3A). ERK1/2 phosphorylation was also increased by EGF (0–100 ng/ml) in a concentration-dependent manner (EC_50_ = 0.23 ng/ml, Figure 3B). Thus, these results are consistent with the well-established role of EGFR in ERK1/2 signaling (35).

When COS-7 cells expressing HA-CRF_1_R were pretreated with the EGFR tyrosine kinase inhibitor AG1478 (100 nM, 30 min), a significant inhibition (~80%) of CRF-induced maximal ERK1/2 phosphorylation was observed (Figure 3C). A concentration-dependent inhibition was observed with AG1478 concentrations of 0–1,000 nM with an IC_50_ of 10 nM (Figure 3D). Importantly, phosphorylation of the EGFR at Tyr^1068^ was detected with Western blots in COS-7 cells beginning at 2 min and becoming maximal at 5–10 min of CRF exposure (100 nM) (Figure 3E). Tyr^1173^ of the EGFR was phosphorylated in parallel with Tyr^1068^ in COS-7 cells stimulated with CRF (Supplementary Figure S5). Together, these results indicate that CRF-activated CRF_1_R triggers phosphorylation of two critical amino acids located within the autophosphorylation loop that are required for EGFR activation (36, 37). Thus, CRF_1_R signaling rapidly transactivates the EGFR, in agreement with a study reporting that Ucn1 stimulated EGFR transactivation in CRF_1_R-expressing HEK293 cells (5).

**Figure 4:**
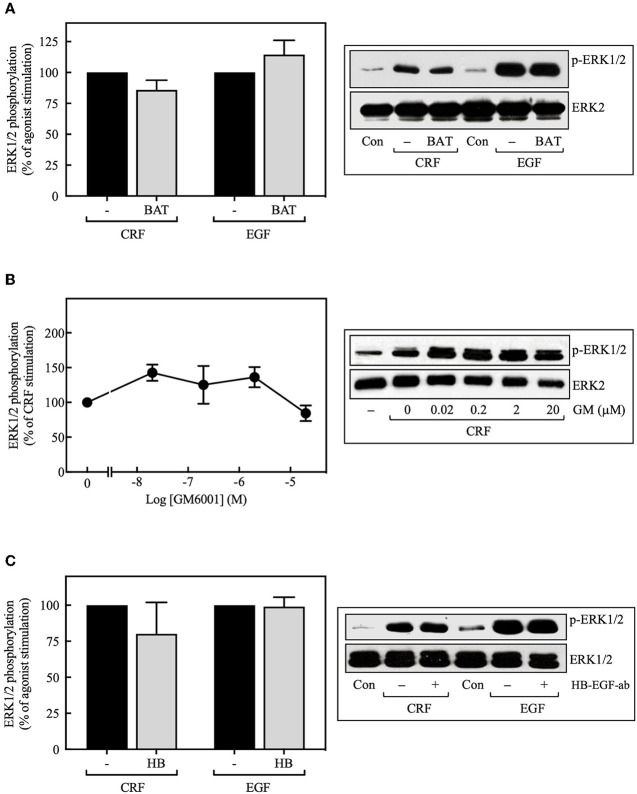
Effect of the MMP inhibitors and the HB-EGF antibody on CRF-stimulated ERK1/2 phosphorylation. COS-7 cells expressing HA-CRF_1_Rs were pretreated with 5 μM batimastat (BAT) **(A)**, the indicated concentrations of GM6001 (GM) **(B)** or 5 μg/ml HB-EGF antibody (HB) **(C)** for 30 min, before stimulation with 100 nM CRF for 5 min **(A–C)** or 10 ng/ml EGF for 10 min **(A,C)**. Total cell lysates were separated by SDS-PAGE and analyzed by immunoblotting with anti-p-ERK1/2 Thr^202^/Tyr^204^, as described in Materials and Methods. ERK1/2 phosphorylation was quantitated by densitometry, and mean values were plotted from three to five independent experiments. Vertical lines represent the S.E.M. Western blots were also probed for total ERK showing equal loading.

MMPs catalyze the release of extracellular heparin-binding EGF (HB-EGF) ligand, which, in turn, binds to and activates the EGFR, thereby stimulating ERK1/2 phosphorylation (38–40). Although this process represents a significant mechanism for GPCR-mediated EGFR transactivation, we found that basal and CRF-stimulated ERK1/2 phosphorylation in transfected COS-7 cells was not altered by inhibiting the formation of the ligand HB-EGF with broad-spectrum MMP inhibitors batimastat BB-94 (5 μM) (Figure 4A) or GM6001 (0–20 μM) (Figure 4B). Similarly, blocking HB-EGF binding to the EGFR with an HB-EGF antibody (5 μg/ml) also failed to inhibit CRF-stimulated ERK1/2 phosphorylation (Figure 4C). In agreement with previous reports (39, 41), GM6001 pretreatment significantly attenuated ERK1/2 phosphorylation induced by PMA but not EGF (Supplementary Figure S6). Altogether, these results exclude a role of MMP in CRF-induced transactivation of the EGFR and subsequent phosphorylation of ERK1/2 in COS-7 cells.

### Src Mediation of CRF_1_R ERK1/2 Signaling

**Figure 5:**
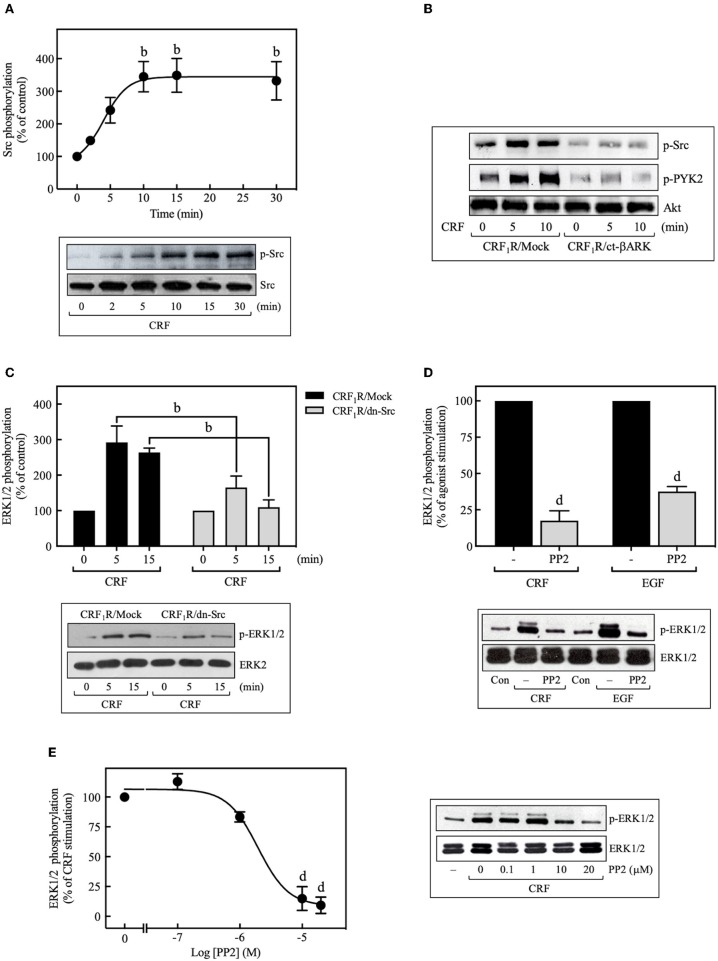
Role of Src tyrosine kinase in CRF-stimulated ERK1/2 phosphorylation. **(A)** COS-7 cells expressing HA-CRF_1_Rs were stimulated with 100 nM CRF for the indicated times. **(B)** COS-7 cells co-transfected with a plasmid pRK5 encoding the carboxyl terminus of βARK that contains its βγ-binding domain (ct-βARK) or an empty control vector (Mock) and the pcDNA3-HA-CRF_1_R expression vector were stimulated with 100 nM CRF for 5 or 10 min. **(C)** COS-7 cells co-transfected with a dominant-negative SrcYF-KM (dn-Src) expression vector or an empty control vector (Mock) and the pcDNA3-HA-CRF_1_R expression vector were stimulated with 100 nM CRF for 5 or 15 min. Cells were pretreated with 10 μM or the indicated concentrations of PP2 for 30 min before stimulation with 100 nM CRF (5 min) **(D,E)** or 10 ng/ml EGF (10 min) **(D)**. Total cell lysates were separated by SDS-PAGE and analyzed by immunoblotting with anti-p-Src Tyr^416^ **(A,B)** or anti-p-ERK1/2 Thr^202^/Tyr^204^ **(C–E)**, as described in Materials and Methods. Src, PYK2, and ERK1/2 phosphorylation were quantitated by densitometry, and mean values were plotted from three to five independent experiments. Vertical lines represent the S.E.M. Western blots were also probed for total Src, total Akt, and total ERK showing equal loading. **(A)** ^b^*p* < 0.01 vs. 0 min. **(C)** ^b^*p* < 0.01 vs. CRF_1_R/Mock (5 or 15 min). **(D)** ^d^*p* < 0.0001 vs. CRF (–), ^d^*p* < 0.0001 vs. EGF (–). **(E)** ^d^*p* < 0.0001 vs. 0 M.

We then investigated the role of Src kinase, which can serve as an important upstream regulator of GPCR signaling via the ERK1/2 cascade (42, 43). Importantly, 100 nM CRF caused marked phosphorylation of Src at Tyr^416^, which is a requirement for Src activation (44), reaching a maximum at 10 min (~3.5-fold increase over time 0), and persisting for more than 30 min (Figure 5A). This activation was dependent on Gβγ release since ct-βARK expression reduced the CRF-induced Src phosphorylation (Figure 5B), and as expected, CRF-induced Src phosphorylation was prevented by pretreatment with the selective Src family kinase inhibitor PP2 (**Figure 9B**). To further evaluate the role of Src in CRF_1_R ERK1/2 signaling, COS-7 cells were co-transfected with the CRF_1_R and a dn-Src. Overexpression of inactive Src prevented ERK1/2 activation by CRF (Figure 5C). Other experiments demonstrated that PP2 pretreatment abolished CRF-stimulated ERK1/2 phosphorylation (Figure 5D), in a concentration-dependent manner (0–20 μM, IC_50_ = 2 μM) (Figure 5E). These findings support our hypothesis that Src plays a central role in CRF_1_R ERK1/2 signaling.

**Figure 6:**
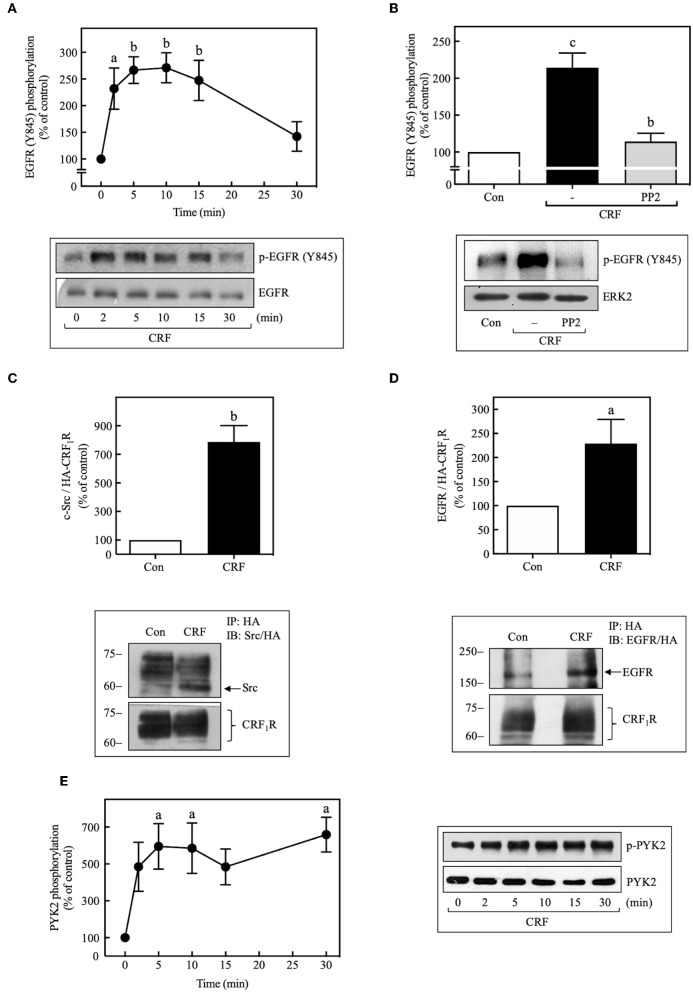
CRF mediates EGFR phosphorylation at Tyr^845^ and activation of PYK2. COS-7 cells expressing HA-CRF_1_Rs were stimulated with 100 nM CRF for the indicated times **(A,E)** or pretreated with 10 μM PP2 for 30 min before stimulation with 100 nM CRF for 5 min **(B)**. Total cell lysates were separated by SDS-PAGE and analyzed by immunoblotting with anti-p-EGFR Tyr^845^ **(A,B)** or anti-p-PYK2 Tyr^402^ **(E)**, as described in Materials and Methods. EGFR and ERK1/2 phosphorylation were quantitated by densitometry, and mean values were plotted from three to five independent experiments. Vertical lines represent the S.E.M. Western blots were also probed for total EGFR, ERK, or PYK2 showing equal loading. **(A)** ^a^*p* < 0.05, ^b^*p* < 0.01 vs. 0 min. **(B)** ^c^*p* < 0.001 vs. Con, ^b^*p* < 0.01 vs. CRF (–). **(E)** ^a^*p* < 0.05 vs. 0 min. **(D)** COS-7 cells expressing HA-CRF_1_Rs were stimulated with 100 nM CRF for 10 min. Total cell lysates were immunoprecipitated with anti-HA antibody and immunoblotted with anti-EGFR antibody or anti-HA antibody. **(C)** Blots were also stripped and reprobed with anti-Src polyclonal antibody. Src and EGFR were quantitated by densitometry, and mean values were plotted from three independent experiments. Vertical lines represent the S.E.M. **(C)** ^b^*p* < 0.01 vs. Con. **(D)** ^a^*p* < 0.05 vs. Con.

We next determined if CRF-stimulated Src activation is required for CRF_1_R-induced transactivation of EGFRs. In this context, previous research has established that Src can activate EGFR signaling by phosphorylating Tyr^845^ of the EGFR protein (45, 46). As shown in Figure 6A, we found that 100 nM CRF stimulated in a time-dependent manner marked phosphorylation of EGFR at Tyr^845^ beginning at 2 min and becoming maximal at 10 min. This effect was blocked by pretreatment of the cells with PP2 (Figure 6B). In a recent study by Perkovska et al. (47), it was shown that V_1b_ vasopressin receptor interacts with Src at basal state, suggesting the formation of a GPCR/Src complex that facilitates MAP kinase activation. To evaluate if a CRF_1_R/Src complex exists under basal conditions, we analyzed CRF_1_R immunoprecipitates for the presence of co-precipitated Src under basal and CRF-stimulated conditions. As shown in Figure 6C, 100 nM CRF induced a robust interaction between the CRF_1_R and Src after 10 min stimulation (~8.0-fold increase over control). Interestingly, it was also observed that under the same immunoprecipitation conditions, the EGFR is also present in the CRF_1_R/co-precipitated complex, even in the absence of CRF stimulation (Figure 6D). After stimulation with 100 nM CRF for 10 min, we observed a significant increase in the CRF_1_R/EGFR interaction (~2.5-fold increase over control). These observations suggest that CRF promotes the formation of a multiprotein complex that would allow rapid EGFR phosphorylation at Tyr^845^ by Src, present in this complex.

It has been shown that, in parallel to Src activation by many GPCRs, the proline-rich tyrosine kinase 2, PYK2, is also phosphorylated and activated, and in association with Src is required for the subsequent transactivation of EGFR (44, 48). Therefore, we decided to assess whether activation of CRF_1_R leads to PYK2 phosphorylation in COS-7 cells. As shown in Figure 6E, 100 nM CRF caused rapid phosphorylation of PYK2 in a time-dependent manner (0–30 min), reaching a maximum effect at 5 min and persisting for at least 30 min of stimulation. Interestingly, and as expected, CRF-mediated PYK2 phosphorylation was also dependent on Gβγ release (Figure 5B).

### PI3K Mediation of CRF_1_R ERK1/2 and Akt Signaling

**Figure 7:**
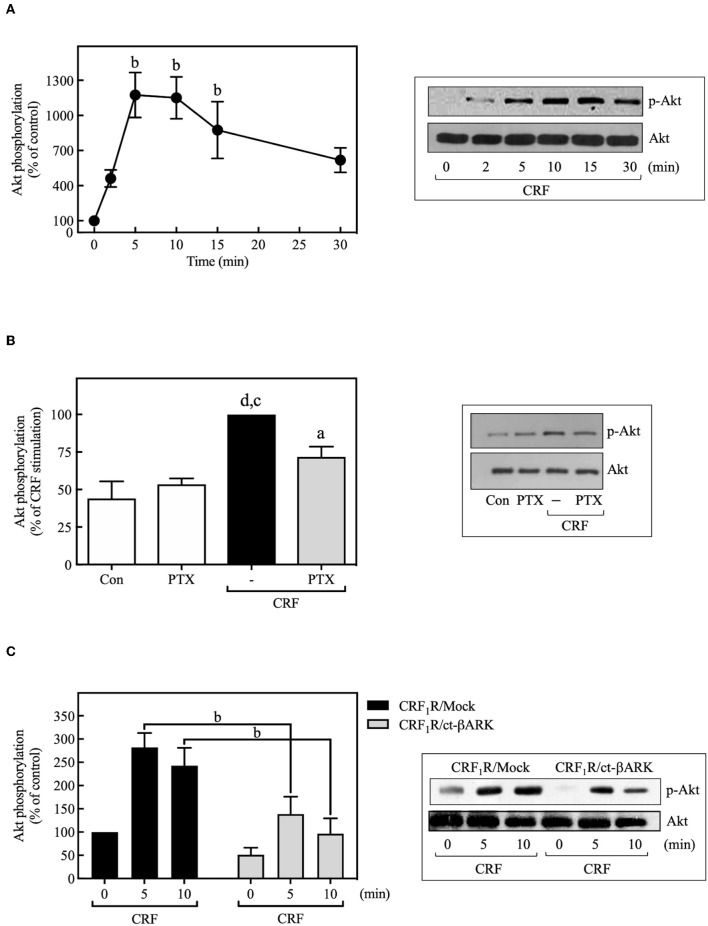
CRF mediates Akt activation. **(A)** COS-7 cells expressing HA-CRF_1_Rs were stimulated with 100 nM CRF for the indicated times. **(B)** COS-7 cells expressing HA-CRF_1_Rs were pretreated with 100 ng/ml PTX for 15 h before stimulation with 100 nM CRF for 5 min. **(C)** COS-7 cells co-transfected with a plasmid pRK5 encoding the carboxyl terminus of βARK that contains its βγ-binding domain (ct-βARK) or an empty control vector (Mock) and the pcDNA3-HA-CRF_1_R expression vector were stimulated with 100 nM CRF for the indicated times. Total cell lysates were separated by SDS-PAGE and analyzed by immunoblotting with anti-p-Akt Ser^473^, as described in Materials and Methods. Akt phosphorylation was quantitated by densitometry, and mean values were plotted from three to five independent experiments. Vertical lines represent the S.E.M. Western blots were also probed for total Akt showing equal loading. **(A)** ^b^*p* < 0.01 vs. 0 min. **(B)** ^d^*p* < 0.0001 vs. Con, ^c^*p* < 0.001 vs. PTX; ^a^*p* < 0.05 vs. CRF (–). **(C)** ^b^*p* < 0.01 vs. CRF_1_R/Mock (5 and 10 min).

PI3Ks can mediate important biological actions of GPCRs, including cell proliferation or survival, by serving as an upstream regulator of Akt and ERK cascades (49, 50). As shown in Figure 7A, 100 nM CRF caused rapid phosphorylation of Akt, an effect that was decreased by PTX pretreatment (Figure 7B) or ct-βARK overexpression (Figure 7C), similar to the previously observed effect on the CRF-induced ERK1/2 phosphorylation, suggesting the participation of G_i_ protein and Gβγ subunits in this process. It is important to note that none of the observed effects of PTX and ct-βARK on CRF actions were present on EGF-stimulated ERK1/2 and Akt phosphorylation (Supplementary Figure S7).

**Figure 8:**
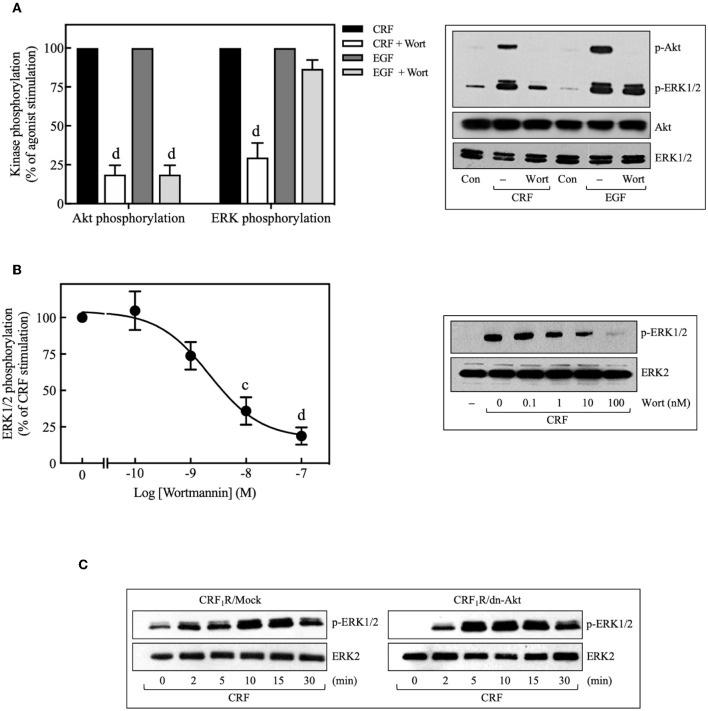
Involvement of the PI3K pathway in CRF-stimulated ERK1/2 phosphorylation. **(A,B)** COS-7 cells expressing HA-CRF_1_Rs were pretreated with 100 nM or the indicated concentrations of wortmannin (Wort) for 30 min before stimulation with 100 nM CRF for 5 min **(A,B)** or 10 ng/ml EGF for 10 min **(A)**. **(C)** COS-7 cells co-transfected with a dominant-negative Akt (K179M) expression vector (dn-Akt) or an empty control vector (Mock) and the pcDNA3-HA-CRF_1_R expression vector were stimulated with 100 nM CRF for the indicated times. Total cell lysates were separated by SDS-PAGE and analyzed by immunoblotting with anti-p-Akt Ser^473^ **(A)** or anti-p-ERK1/2 Thr^202^/Tyr^204^ **(A–C)**, as described in Materials and Methods. Akt and ERK1/2 phosphorylation were quantitated by densitometry, and mean values were plotted from three to five independent experiments. Vertical lines represent the S.E.M. Western blots were also probed for total Akt or ERK showing equal loading. **(A)** ^d^*p* < 0.0001 vs. CRF (p-Akt), ^d^*p* < 0.0001 vs. EGF (p-Akt); ^d^*p* < 0.0001 vs. CRF (p-ERK), **(B)** ^c^*p* < 0.001, ^d^*p* < 0.0001 vs. 0 M.

**Figure 9:**
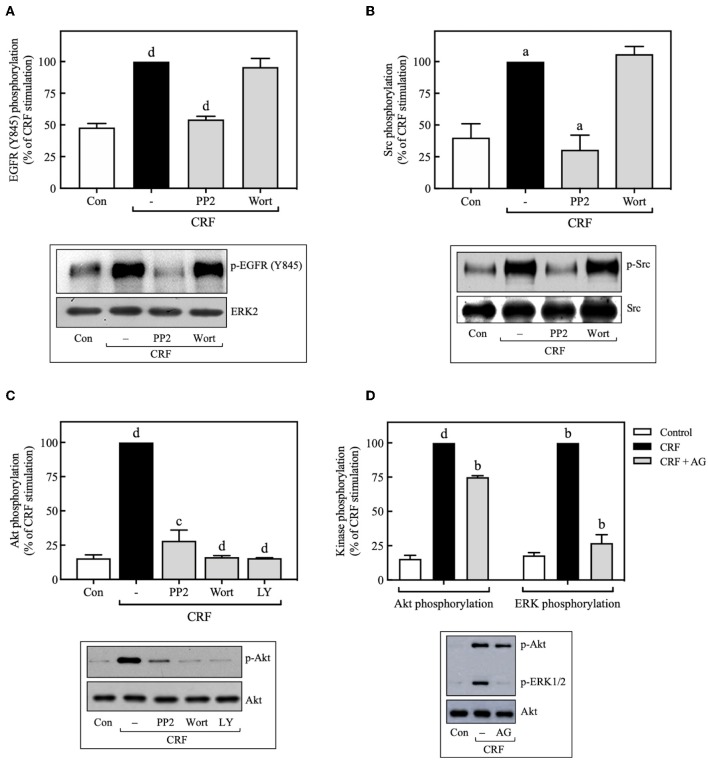
Src is an upstream regulator of EGFR and PI3K. COS-7 cells expressing HA-CRF_1_Rs were pretreated with 10 μM PP2, 100 nM wortmannin (Wort) **(A–C)** or 10 μM LY294002 (LY) **(C)** for 30 min, before stimulation with 100 nM CRF for 5 min. **(D)** Effect of 100 nM AG1478 (AG) on CRF- (100 nM, 5 min) induced ERK1/2 and Akt phosphorylation. Total cell lysates were separated by SDS-PAGE and analyzed by immunoblotting with anti-p-EGFR Tyr^845^ **(A)**, anti-p-Src Tyr^416^ **(B)**, anti-p-Akt Ser^473^ **(C,D)** or anti-p-ERK1/2 Thr^202^/Tyr^204^ **(D)**, as described in Materials and Methods. EGFR, Src, Akt, and ERK1/2 phosphorylation were quantitated by densitometry, and mean values were plotted from three to five independent experiments. Vertical lines represent the S.E.M. Western blots were also probed for total ERK, Src or Akt showing equal loading. **(A)** ^d^*p* < 0.0001 vs. Con; ^d^*p* < 0.0001 vs. CRF (–). **(B)** ^a^*p* < 0.05 vs. Con; ^a^*p* < 0.05 CRF(–). **(C)** ^d^*p* < 0.0001 vs. Con; ^c^*p* < 0.001, ^d^*p* < 0.0001 vs. CRF (–). **(D)** ^d^*p* < 0.0001 vs. Con (p-Akt), ^b^*p* < 0.01 vs. CRF (p-Akt); ^b^*p* < 0.01 vs. Con (p-ERK), ^b^*p* < 0.01 vs. CRF (p-ERK).

Pretreatment with selective PI3K inhibitors, wortmannin (100 nM) (Figure 8A), or LY294002 (10 μM) (Figure 9C) abolished CRF_1_R-mediated Akt signaling activation. Similarly, inhibition of PI3K by 100 nM wortmannin abolished the stimulatory action of EGF on Akt (Figure 8A), thereby demonstrating that the PI3K pathway is required for both CRF- and EGF-induced Akt phosphorylation. Considering that an upstream PI3K mechanism can also regulate CRF_1_R and CRF_2_R signaling via the ERK1/2 cascade in A7r5, CATH.a, and transfected CHO cells (9, 12), we investigated the potential role of PI3K in the activation of ERK1/2 by HA-CRF_1_Rs expressed in COS-7 cells. In this context, activation of RTKs, such as the EGFRs, has been shown to recruit PI3K and activate ERK1/2 (50–53). However, contradictory data on PI3K involvement in EGFR-induced ERK1/2 phosphorylation have been reported (54–56). In this regard, to find out if EGF-mediated ERK1/2 phosphorylation observed in COS-7 cells is depending on PI3K activation, we analyze the effect of 100 nM wortmannin on the EGF ERK1/2 activation. As shown in Figure 8A, pretreatment with wortmannin was unable to inhibit the effect of EGF, suggesting that PI3K does not participate in this mechanism. In contrast, pretreatment with wortmannin abolished CRF-stimulated ERK1/2 phosphorylation (Figure 8A) in a concentration-dependent manner (0–100 nM), confirming an intermediary role for PI3K in CRF_1_R ERK signaling (Figure 8B). To examine the contribution of CRF-mediated activation of Akt to the phosphorylation of ERK1/2, we evaluated the effect of the dn-Akt mutant. As shown in Figure 8C, overexpression of dn-Akt had no significant effect on ERK1/2 activation after stimulation with CRF (Figure 8C), suggesting that Akt does not participate in the activation of ERK1/2 by CRF. Because in the present work we do not show evidence about impairment of kinase activity of the dn-Akt, it will be necessary the use of other approaches, such as genetic tools or inhibitors, to provide more evidence regarding the possible no effect of Akt on the ERK 1/2 pathway. Because in the present work we do not show evidence about impairment of kinase activity of the dn-Akt, it will be necessary the use of other approaches, such as genetic tools or inhibitors, to provide more evidence regarding the possible Akt lack of effect on ERK 1/2 pathway. Consequently, our results suggest that PI3K can regulate the transduction of CRF_1_R signals through the ERK cascade, possibly independently of Akt.

### Src Acts Upstream and PI3K Downstream of the EGFR During CRF-Induced ERK1/2 Activation

Since we found that CRF-induced EGFR transactivation mediates ERK1/2 phosphorylation through Src- and PI3K-dependent mechanisms, we next determined if CRF-induced PI3K activation occurs upstream or downstream of Src and EGFR. Wortmannin inhibition of PI3K had no effect on CRF-induced phosphorylation of EGFR at Tyr^845^ (Figure 9A), suggesting that PI3K acts downstream of Src and EGFR. Consistent with these results, we also observed that wortmannin pretreatment did not alter CRF-induced phosphorylation of Src at Tyr^416^ (Figure 9B). Therefore, CRF_1_R-stimulated transactivation of EGFR and phosphorylation of ERK1/2 mediated by Src was independent of PI3K. It has been reported that the PI3K/Akt signaling pathway can be activated at least by two independent mechanisms: (i) EGFR transactivation (57), and (ii) upstream Src activation (58, 59). We observed that CRF-induced Akt activation was completely inhibited by the Src inhibitor, PP2 (Figure 9C), suggesting that Src is an upstream regulator of PI3K and Akt. We next measured the effect of the specific EGFR tyrosine kinase inhibitor, tyrphostin AG1478, on CRF-stimulated Akt phosphorylation. As observed in Figure 9D, while CRF-induced ERK1/2 activation was totally dependent on EGFR transactivation (Figure 9D), Akt phosphorylation was only partially dependent. Thus, we hypothesize that PI3K/Akt pathway signaling by CRF_1_R may involve two mechanisms: (i) a strong dependence on upstream Src activating PI3K and then Akt (Figure 9C); (ii) a weak dependence on EGFR transactivation (Figure 9D).

### Role of β-Arrestin-2 in the CRF-Mediated ERK1/2 Activation

**Figure 10:**
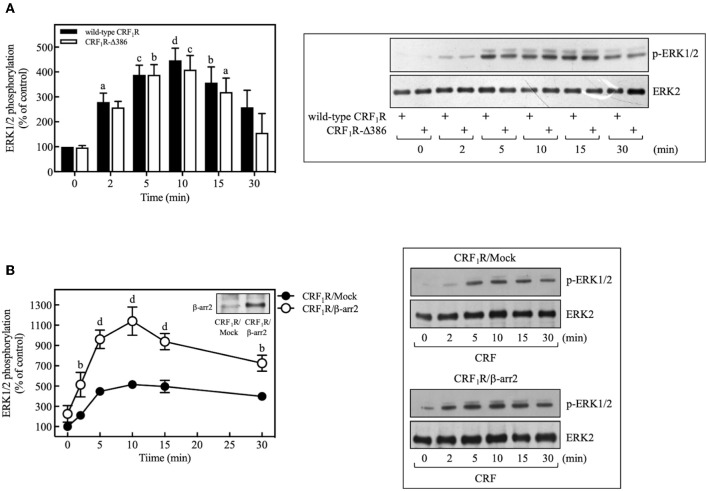
β-arrestin does not participate in the CRF-stimulated ERK 1/2 phosphorylation in COS-7 cells. **(A)** COS-7 cells expressing HA-CRF_1_R (wild-type CRF_1_R) or HA-CRF_1_R-Δ386 mutant were stimulated with 100 nM CRF for the indicated times. **(B)** COS-7 cells co-transfected with a β-arrestin-2 (β-arr2) expression vector or an empty control vector (Mock) and the pcDNA3-HA-CRF_1_R expression vector were stimulated with 100 nM CRF for the indicated times. Total cell lysates were separated by SDS-PAGE and analyzed by immunoblotting with anti-p-ERK1/2 Thr^202^/Tyr^204^, as described in Materials and Methods. ERK1/2 phosphorylation was quantitated by densitometry, and mean values were plotted from three to five independent experiments. Vertical lines represent the S.E.M. Western blots were also probed for total ERK showing equal loading. **(A)** ^a^*p* < 0.05, ^c^*p* < 0.001, ^d^*p* < 0.0001, ^b^*p* < 0.01 vs. 0 min (CRF_1_R); ^b^*p* < 0.01, ^c^*p* < 0.001, ^a^*p* < 0.05 vs. 0 min (CRF_1_R-Δ386). **(B)** ^b^*p* < 0.01 vs. CRF_1_R (2 or 30 min); ^d^*p* < 0.0001 vs. CRF_1_R (5, 10, or 15 min).

In recent years, it has been identified that β-arrestin proteins play an important role in mediating the actions of GPCRs, particularly those related to activation and regulation of Src and mitogenic pathways, in particular, the ERK1/2 signaling cascade (60). To determine the role of β-arrestins in the CRF-mediated ERK1/2 activation observed above, we used a phosphorylation-deficient mutant CRF_1_R, which also shows a diminished agonist-dependent β-arrestin-2 recruitment (24). As shown in Figure 10A, COS-7 cells transiently transfected with HA-CRF_1_R-Δ386 mutant showed a similar response in ERK1/2 phosphorylation compared to that observed with CRF_1_R. The apparent independence of CRF-mediated activation of ERK1/2 from β-arrestin-2 could be explained by the low β-arrestin expression level previously detected in COS-7 cells (61, 62). To assess this possibility, we evaluated the effect of β-arrestin-2 overexpression in COS-7 cells, since CRF_1_R activation has been shown to lead to selective recruitment of β-arrestin-2 in both HEK293 cells and neurons (24, 63). As observed in Figure 10B, cells co-expressing HA-CRF_1_R and β-arrestin-2 showed a significant increase in the CRF-mediated ERK1/2 phosphorylation, suggesting that β-arrestin involvement in CRF_1_R ERK1/2 signaling depends on its cellular expression levels.

## Discussion

In the present study, we investigated the molecular mechanisms associated with the activation of ERK1/2 and Akt signaling cascades by the human CRF_1_R in COS-7 cells. Our data suggest that agonist-stimulated CRF_1_R promotes G_i_ activation and Gβγ release which, in turn, stimulate phosphorylation and activation of Src kinase. Once Src is active, it mediates ERK1/2 phosphorylation by at least two independent signaling mechanisms: (i) phosphorylation and transactivation of the EGFR, (ii) activation of PI3K. Interestingly, CRF_1_R-induced Akt phosphorylation also requires Src-mediated activation of PI3K as the main mechanism, but it is mostly independent of EGFR transactivation.

Defining the molecular mechanisms for ERK1/2 signaling by a GPCR has become a significant focus of signal transduction research due to the multifaceted pathways mediating signaling via the ERK1/2-MAP kinase cascade. A significant role of the ERK1/2-MAP kinase pathway has been recognized in the biological action of both CRF_1_R and CRF_2_R. ERK1/2 is widely distributed in the brain and is considered an essential regulator of the molecular processes involved in response to stress (6, 64). It is well-established that most GPCRs signal *via* ERK1/2-MAP kinase cascades through distinct G_i_-, G_s_-, and G_q_-dependent signaling pathways. In the case of the CRF_1_R, it has been identified that the G_s_/PKA pathway is importantly involved in the activation of MAP kinase cascades (12, 15, 18). In contrast, we found that pretreating CRF_1_R-expressing COS-7 cells with PKA inhibitors H89 or Rp-cAMP did not alter the ability of CRF to stimulate ERK1/2 phosphorylation. Although earlier research proposed that high cellular expression of the serine-threonine kinase B-Raf molecularly switches “upstream” ERK1/2 activation by G_s_-coupled GPCRs to a PKA mechanism (14), pretreating fetal hippocampal cells with the PKA inhibitor H89 only produced a small reduction in CRF_1_R-mediated ERK phosphorylation despite very high hippocampal levels of B-Raf (18). Furthermore, H89 failed to inhibit CRF_1_R-mediated ERK signaling in brain-derived CATH.a, rat fetal microglial, locus coeruleus, and transfected CHO cells (8, 9, 12, 17). In fact, ERK activation by CRF_1_R in HEK293 cells was markedly decreased after the third intracellular loop's Ser^301^ was phosphorylated by PKA (65). Thus, a cAMP-dependent PKA → Rap1 → B-Raf mechanism does not always mediate ERK1/2 signaling by G_s_-coupled receptors. EPAC, a guanine nucleotide exchange factor that is activated by intracellular cAMP, has been shown to regulate activation of Rap1 and ERK1/2 without the involvement of PKA (66). G_s_-coupled CRF_1_R signaling can stimulate ERK1/2 phosphorylation by activating upstream EPAC2 independent of PKA in certain cell lines (17, 67). Interestingly, neither Epac nor PKA was found to mediate Akt cascade signaling by CRF_2(b)_R in HEK293 (21).

The versatility of the CRF_1_R to activate different signaling pathways has allowed its coupling to G_q_ proteins to be identified (4). G_q_ conveys a signal to activate PKC which then triggers MAP kinase cascades. Thus, it has been shown that G_q_/PLC/PKC cascade signaling by CRF_1_R activated by Ucn1 contributes to phosphorylation of ERK1/2 in CRF_1_R-expressing myometrial, CHO, HEK293, and rat hippocampal cells (12, 13, 18). However, in pituitary AtT20 cells and CATH.a cells, PKC is not involved in Ucn1-stimulated ERK1/2 phosphorylation (12). In our study, pretreatment with the PKC inhibitor, BIM, increased rather than reduced CRF-stimulated ERK1/2 phosphorylation, suggesting that PKC may negatively regulate CRF_1_R ERK1/2 signaling in COS-7 cells, although the specific mechanism for this effect remains to be determined.

The use of PTX in our study suggests the participation of G_i_ protein in the CRF-dependent activation of ERK and Akt pathways. Interestingly, it is now well-established that during GPCR/G_i_ signaling, Gβγ release can activate a myriad of effectors to modulate diverse signaling pathways downstream of GPCRs, including Src, which in turn activate EGFR to promote ERK1/2 activation (43, 68, 69). Gβγ-activated Src can also associate PYK2. When we blocked that action of Gβγ subunits in COS-7 cells by overexpressing the ct-βARK peptide, which is a Gβγ subunit scavenger (70, 71), CRF-stimulated ERK phosphorylation was decreased by ~40%. Moreover, ct-βARK overexpression markedly reduced phosphorylation of Src and Akt. In agreement, another group has also found that CRF_1_R ERK1/2 signaling is only partially dependent on Gβγ, although their study did not assess the role of Gβγ subunits in the activation of upstream ERK1/2 pathways. Differences in CRF_1_R-mediated activation of the ERK1/2-MAP kinase cascade are probably attributable to variations in the signaling properties of transfected CRF_1_Rs expressed in different cell lines utilized in these studies. We are presently investigating other upstream factors including β-arrestins that regulate Src and EGFR mediation of CRF_1_R ERK1/2 signaling.

Our experiments did demonstrate that CRF-stimulated phosphorylation of ERK1/2 and EGFR occurred in parallel, while pretreatment with the EGFR kinase inhibitor, AG1478, caused a concentration-dependent inhibition of CRF-stimulated ERK1/2 phosphorylation. In agreement, it has been shown that EGFR transactivation is required for Ucn1-stimulated ERK1/2 phosphorylation in transfected HEK293 cells (5). In contrast to our data indicating that a MMP/HB-EGF ligand mechanism was not involved, however, this group reported that MMP generation of an HB-EGF ligand transactivated the EGFR during CRF_1_R ERK1/2 signaling (5). Therefore, EGFR transactivation can play a critical role in CRF_1_R signaling via the ERK1/2-MAP kinase cascade.

Earlier studies have implicated a PI3K-dependent mechanism in CRF_1_R ERK1/2 signaling based on the observation that pretreatment with PI3K inhibitors attenuated sauvagine- and Ucn1-stimulated ERK1/2 phosphorylation in CRF_1_R-expressing CHO and HEK293 cells (5, 9, 12). PI3K is also involved in CRF_2(b)_R-stimulated ERK1/2 activation in CHO, A7r5, and mouse neonatal cardiomyocyte cells (12, 19). However, the activation sequence of PI3K, EGFR, and ERK1/2 during CRF_1_R signaling has not been fully elucidated. Here we observed that pretreating CRF_1_R-expressing COS-7 cells with the PI3K inhibitors wortmannin and LY294002 inhibited CRF-stimulated phosphorylation of ERK1/2 and Akt. Previous studies suggest that PI3K activity is required for Gβγ-mediated MAP kinase signaling pathway at a point upstream of Sos and Ras activation (50, 72).

Because we also found that AG1478 abolished phosphorylation of ERK1/2 while only decreasing Akt phosphorylation 25% in transfected COS-7 cells stimulated with CRF, upstream activation of the PI3K/Akt pathway by CRF_1_R is not strongly dependent on EGFR transactivation. In this context, our study suggests that Src acts as a critical mediator of PI3K activation, independent of EGFR transactivation, which, in turn, stimulates Akt and ERK1/2 phosphorylation. Previous studies have shown that activated Src directly associates with PI3K through interaction between the SH3 domain of Src and the proline-rich motif in the p85 regulatory subunit of PI3K, thereby increasing the specific activity of PI3K (59). Furthermore, intermediary proteins have also been identified to mediate Src-induced PI3K activation, such as p66Shc, Rap1, and FAK. Thus, our study raises the possibility that Src activates PI3K, although the specific mechanism for this effect remains to be determined.

**Figure 11:**
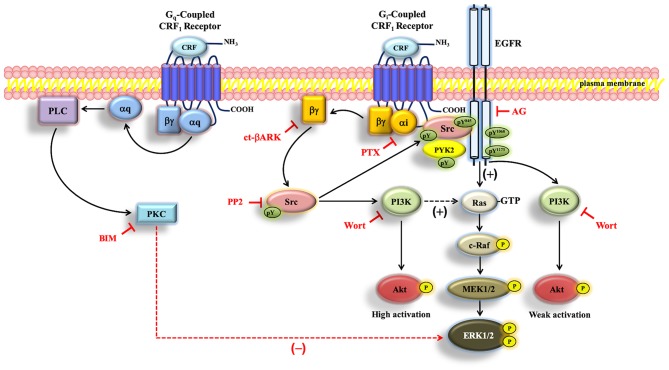
Schematic representation of the proposed CRF-mediated ERK1/2/Akt signaling mechanism in CRF_1_R transfected COS-7 cells. When COS-7 cells are stimulated with CRF, the overexpressed CRF_1_R leads to activation of G_i_ protein and the subsequent dissociation of α_i_-GTP and βγ. Gβγ subunit is able to activate Src, which plays a central role in the activation of EGFR, through the formation of a protein complex that contains CRF_1_R, EGFR, and Src. PI3K, independently of Akt, is involved in ERK1/2 activation, presumably through Ras/C-Raf/MEK1/2. On the other hand, Src/PYK2 transactivates EGFR. Such EGFR transactivation leads to activation of the MAP kinase/ERK1/2 cascade and parallelly to weak activation of the PI3K/Akt pathway. Solid arrows indicate signaling mechanisms that have been identified. Dashed arrows indicate that the precise mechanism associated with the CRF-mediated regulation of Ras by PI3K and ERK1/2 by PKC remains to be determined. Plus sign (+) means positive regulation, and minus sign (–) means negative regulation of the ERK pathway.

For certain GPCRs, Src has been shown to induce EGFR transactivation, stimulate the PI3K-Akt pathway, and activate the ERK1/2 cascade (43, 48, 70). A novel finding in our study is the rapid and parallel phosphorylation of Src, PYK2, the EGFR, Akt, and ERK1/2 in CRF_1_R-expressing COS-7 cells stimulated with CRF. Importantly, we demonstrated that inhibiting Src function with PP2 markedly reduced or abolished the CRF-stimulated activation of Src, PYK2, the EGFR, and ERK1/2, suggesting that Src has a central role in regulating CRF_1_R ERK1/2 signaling. Thus, our results clearly show that Src triggers signal transduction by two important pathways culminating in ERK activation by CRF_1_R: (i) EGFR activation of the classical Ras/Raf/MEK/ERK pathway, and (ii) PI3K regulation and subsequent activation of ERK1/2 (Figure 11).

To the best of our knowledge, our study demonstrates for the first time that Src regulates ERK and Akt signaling by the CRF_1_R. Yuan et al. (20) reported that Src was an upstream regulator of ERK signaling by both the CRF_1_R and CRF_2_R in the mouse atrial HL-1 cardiomyocytes cell line based on the effects of antalarmin (a CRF_1_R antagonist) and anti-sauvagine (a CRF_2_R antagonist). Although CRF_1_R was reported to be expressed in the human heart (73, 74), Ikeda et al. (75) reported that CRF_2(b)_R is the major CRF receptor expressed in the HL-1 mouse atrial cardiomyocyte cell line with no measurable level of CRF_1_R mRNA. Their data detecting only CRF_2_R expression in HL-1 cells is consistent with previous and more recent studies demonstrating only CRF_2_R expression in rat and mouse cardiomyocytes (19, 76, 77). Therefore, ERK1/2 signaling stimulated by Ucns in cardiomyocytes is mediated through CRF_2_R, which appears to be the main mediator of the cardiac stress response (78, 79), rather than through CRF_1_R. Additionally, recent observations also indicate that CRF_2_R controls the cellular organization and colon cancer progression, specifically through the Src/ERK pathway (80, 81). Thus, while all previous findings are relevant to CRF_2_Rs, our findings show for the first time that Src plays an important role in the regulation of ERK1/2 and Akt signaling by the CRF_1_R.

An important finding of our study was the detection of a signaling protein scaffold, which contains CRF_1_R, Src, and EGFR (Figures 6C,D). While the association between CRF_1_R and Src was totally dependent on CRF agonist activation, a constitutive interaction between CRF_1_R and EGFR was also detected, which was increased after CRF stimulation. In this context, it has previously reported that some GPCRs physically interact with EGFR in the absence of receptor ligands, a condition that may increase the efficiency of EGFR transactivation (29, 82–84). Thus, it is possible that the detected constitutive association between CRF_1_R and EGFR facilitates a more rapid CRF agonist-induced recruitment of Src to the EGFR and subsequent phosphorylation and activation of EGFR. With regard to this possibility, the presence of a putative proline-rich domain-binding SH3 motif (*ProXXPro;* X, any amino acid), located in the carboxyl terminus of the CRF_1_R (*Pro*^398^*Thr*^399^*Ser*^400^*Pro*^401^) may provide a site for the direct interaction between Src and CRF_1_R after agonist stimulation. However, the CRF_1_R-Δ386 mutant, which lacks the *ProXXPro* motif, induces a similar degree of ERK1/2 activation that is induced by the wild-type CRF_1_R, which suggests this putative region may not participate in the binding to Src (Figure 10A).

Moreover, there is evidence that Tyr phosphorylation of GPCRs plays a role in mediating GPCR-Src interactions (43). For instance, in studies conducted in A431 epidermoid carcinoma cells, stimulation of the β_2_-adrenergic receptor (β_2_-AR) with isoproterenol, results in phosphorylation of the receptor on Tyr^305^ (43, 85). The mutation of this residue to Phe abolishes Src/β_2_-AR association and impairs Src activation. This residue lies within a canonical Src SH2 binding domain, and it is proposed that Src directly binds the Tyr-phosphorylated β_2_-AR. Interestingly, the CRF_1_R has also a single putative SH2 binding domain (*TyrXX-hyd*; hyd, hydrophobic amino acid) located at the end of the third intracellular loop (*Tyr*^309^*Arg*^310^*Lys*^311^*Ala*^312^), which may be a site where Src can directly interact with CRF_1_R. Further work is needed to establish the importance of this putative site in the agonist-induced CRF_1_R/Src interaction and Src activation.

β-arrestins are a small family of cytosolic proteins initially identified for their central role in GPCRs desensitization. Furthermore, β-arrestins act as adaptors in clathrin-mediated receptor endocytosis (86). In this sense, their role in CRF_1_R homologous desensitization and endocytosis is well-recognized, particularly for β-arrestin-2 (24, 63, 87). It is now well-established, however, that β-arrestins can also act as GPCR-signaling transducers that recruit and activate many other signaling molecules, including Src, MAP kinase, NF-κB and PI3K that modulate diverse cellular responses (64, 86). β-arrestin regulation of CRF/CRF_1_R signaling is still not fully understood.

Regarding β-arrestin regulation of CRF/CRF_1_R-mediated ERK1/2 activation, β-arrestin-2-mediation of CRF_1_R internalization participates in the late phase of sustained ERK1/2 activation after G protein activation and B-Raf mediate the early phase of ERK1/2 activation (88). However, overexpression of PDS-95 in HEK293 cells, a CRF_1_R-interacting protein, inhibited CRF-induced-CRF_1_R internalization in a PDZ-binding motif-dependent manner by suppressing β-arrestin-2 recruitment. Intriguingly, neither the overexpression of PSD-95 nor the knockdown of endogenous PSD-95 affected CRF-mediated activation of ERK1/2 (89).

Under this experimental evidence and due to the importance of β-arrestins in the scaffolding and activation of Src and regulation of MAP kinase cascades, it was decided to evaluate their role in the CRF/CRF_1_R-mediated ERK1/2 activation observed in COS-7 cells. Using a phosphorylation-deficient mutant CRF_1_R, which has a decreased interaction with β-arrestin-2 (24), no significant changes in the activation of ERK1/2 were detected after agonist stimulation (Figure 10A), suggesting that β-arrestin-2 is not involved in the CRF/CRF_1_R-mediated ERK1/2 activation observed in COS-7. This finding, however, can be explained in part by the low expression level of β-arrestins in COS-7 cells (61, 62). This hypothesis is supported by our data showing that overexpressing β-arrestin-2 in COS-7 notably increased the CRF/CRF_1_R-mediated ERK1/2 activation (Figure 10B). Likewise, β-arrestin overexpression in COS-7 cells has been found to augment CRF_1_R internalization (24). Thus, our data provide evidence about the involvement of β-arrestin-2 in the CRF/CRF_1_R MAP kinase activation in cells with sufficient β-arrestin expression.

Our findings on signaling pathways activated by CRF_1_R help to elucidate the molecular mechanisms involved in response to stress mediated by this receptor. For instance, kinases in the ERK1/2-MAP kinase cascade, including Src and PYK2, are highly expressed in extended amygdala and forebrain neurons regulating anxiety defensive behavior and stress responsiveness (90–92). Acute stress or central CRF administration induces rapid phosphorylation of ERK1/2 in the basolateral amygdala and hippocampal neurons and prominent anxiety-like behavior in rats and mice (93–95). Furthermore, CRF_1_Rs can also signal through other cellular pathways that may be involved in post-traumatic stress disorder pathophysiology. As we showed here, CRF_1_R activated by CRF stimulated rapid phosphorylation of Akt at Ser^473^ that is mediated by upstream Src and PI3K. Preclinical research has shown that activated Akt in the ventral tegmentum promotes resilience to anxiety- and depressive-like responses to stress (3, 96), while high levels of phosphorylated Akt in the dorsal hippocampus and basolateral amygdala prolongs contextual and sensitized fear induced by inescapable stress (3, 97). Therefore, the consequences of CRF_1_R Akt signaling during trauma and severe stress may differ depending on the brain region. Hence, ERK1/2-MAP kinase and Akt cascade signaling by CRF_1_R regulated by Src, PYK2, and EGFR may have critical roles in stress-induced anxiety and depression.

## Conclusions

In summary, the data presented herein establish that the tyrosine kinase Src serves as a central upstream regulator of ERK1/2-MAP kinase and Akt cascade signaling by the human CRF_1_R in COS-7 cells. Although CRF_1_R coupling to G proteins strongly activates PKA and PKC pathways, neither second messenger kinases were involved in CRF_1_R-mediated ERK1/2 signaling. However, Gβγ released during activation of CRF_1_R by CRF, particularly from G_i_, stimulates phosphorylation of Src and PYK2, which in turn promotes transactivation of the EGFR through the formation of a heterotrimeric complex formed by the association of CRF_1_R, Src, and EGFR. EGFR transactivation, which occurred independent of MMP generation of the HB-EGF ligand, was essential for CRF-stimulated ERK1/2 phosphorylation while having only a small role in CRF_1_R-mediated Akt activation. Although PI3K activation contributes to CRF-stimulated ERK1/2 phosphorylation, CRF_1_R-mediated EGFR transactivation is independent of the PI3K/Akt pathway. In contrast, CRF_1_R Akt signaling while also being mediated by generation of Gβγ and phosphorylation of Src is weakly dependent on EGFR transactivation.

## Data Availability Statement

All datasets generated for this study are included in the article/Supplementary Material.

## Author Contributions

JO-R, RH, and KC conceived the project. JO-R, FD, and RH designed the experiments. GP-M, AF-G, JH-A, and MD-C carried out the experiments. JO-R, GP-M, and AF-G analyzed and discussed the data. JO-R and RH wrote the manuscript. All authors read and approved the final version of the manuscript and took a due care to ensure the integrity of the work.

## Dedication

This work is dedicated to Dr. Kevin J. Catt, who was an extraordinary scientist, mentor, and friend who passed away on October 1, 2017.

### Conflict of Interest

FD was employed by Novaliq GmbH. The remaining authors declare that the research was conducted in the absence of any commercial or financial relationships that could be constructed as a potential conflict of interest.

## Abbreviations

CRF: Corticotropin-releasing factor
CRF_1_R: corticotropin-releasing factor receptor type 1
ct-βARK: β-adrenergic receptor kinase carboxyl terminus peptide
EGF: epidermal growth factor
EGFR: epidermal growth factor receptor
ERK1/2: extracellularly regulated kinases 1 and 2
GPCR: G protein-coupled receptor
MMP: matrix metalloproteinase
MAP kinase: mitogen-activated protein kinase
PYK2: proline-rich tyrosine kinase 2
PI3K: phosphatidylinositol 3-kinase
c-Src: human homolog of the v-Src Rous sarcoma proto-oncogene
Ucn: urocortin.

## References

[1] Deussing JM Chen A. The corticotropin-releasing factor family: physiology of the stress response. Physiol Rev. (2018) 98:2225–86. 10.1152/physrev.00042.2017 30109816

[2] Hillhouse EW Grammatopoulos DK. The molecular mechanisms underlying the regulation of the biological activity of corticotropin-releasing hormone receptors: implications for physiology and pathophysiology. Endocr Rev. (2006) 27:260–86. 10.1210/er.2005-0034 16484629

[3] Hauger RL Risbrough V Oakley RH Olivares-Reyes JA Dautzenberg FM. Role of CRF receptor signaling in stress vulnerability, anxiety, and depression. Ann N Y Acad Sci. (2009) 1179:120–43. 10.1111/j.1749-6632.2009.05011.x 19906236 PMC3115623

[4] Dautzenberg FM Gutknecht E Van der Linden I Olivares-Reyes JA Durrenberger F Hauger RL Cell-type specific calcium signaling by corticotropin-releasing factor type 1 (CRF1) and 2a (CRF2(a)) receptors: phospholipase C-mediated responses in human embryonic kidney 293 but not SK-N-MC neuroblastoma cells. Biochem Pharmacol. (2004) 68:1833–44. 10.1016/j.bcp.2004.07.013 15450949

[5] Punn A Levine MA Grammatopoulos DK Identification of signaling molecules mediating corticotropin-releasing hormone-R1alpha-mitogen-activated protein kinase (MAPK) interactions: the critical role of phosphatidylinositol 3-kinase in regulating ERK1/2 but not p38 MAPK activation. Mol Endocrinol. (2006) 20:3179–95. 10.1210/me.2006-0255 16959871

[6] Arzt E Holsboer F. CRF signaling: molecular specificity for drug targeting in the CNS. Trends Pharmacol Sci. (2006) 27:531–8. 10.1016/j.tips.2006.08.007 16935354

[7] Dermitzaki E Tsatsanis C Gravanis A Margioris AN. Corticotropin-releasing hormone induces Fas ligand production and apoptosis in PC12 cells via activation of p38 mitogen-activated protein kinase. J Biol Chem. (2002) 277:12280–7. 10.1074/jbc.M111236200 11790788

[8] Wang W Ji P Dow KE. Corticotropin-releasing hormone induces proliferation and TNF-alpha release in cultured rat microglia *via* MAP kinase signalling pathways. J Neurochem. (2003) 84:189–95. 10.1046/j.1471-4159.2003.01544.x 12485415

[9] Rossant CJ Pinnock RD Hughes J Hall MD McNulty S. Corticotropin-releasing factor type 1 and type 2alpha receptors regulate phosphorylation of calcium/cyclic adenosine 3′,5′-monophosphate response element-binding protein and activation of p42/p44 mitogen-activated protein kinase. Endocrinology. (1999) 140:1525–36. 10.1210/endo.140.4.6656 10098484

[10] Cao J Cetrulo CL Theoharides TC. Corticotropin-releasing hormone induces vascular endothelial growth factor release from human mast cells via the cAMP/protein kinase A/p38 mitogen-activated protein kinase pathway. Mol Pharmacol. (2006) 69:998–1006. 10.1124/mol.105.019539 16332989

[11] Park HJ Kim HJ Lee JH Lee JY Cho BK Kang JS. Corticotropin-releasing hormone (CRH) downregulates interleukin-18 expression in human HaCaT keratinocytes by activation of p38 mitogen-activated protein kinase (MAPK) pathway. J Invest Dermatol. (2005) 124:751–5. 10.1111/j.0022-202X.2005.23656.x 15816833

[12] Brar BK Chen A Perrin MH Vale W. Specificity and regulation of extracellularly regulated kinase1/2 phosphorylation through corticotropin-releasing factor (CRF) receptors 1 and 2beta by the CRF/urocortin family of peptides. Endocrinology. (2004) 145:1718–29. 10.1210/en.2003-1023 14670995

[13] Grammatopoulos DK Randeva HS Levine MA Katsanou ES Hillhouse EW Urocortin, but not corticotropin-releasing hormone (CRH), activates the mitogen-activated protein kinase signal transduction pathway in human pregnant myometrium: an effect mediated *via* R1alpha and R2beta CRH receptor subtypes and stimulation of Gq-proteins. Mol Endocrinol. (2000) 14:2076–91. 10.1210/mend.14.12.0574 11117536

[14] Stork PJS. Does Rap1 deserve a bad Rap? Trends Biochem Sci. (2003) 28:267–75. 10.1016/S0968-0004(03)00087-2 12765839

[15] Kovalovsky D Refojo D Liberman AC Hochbaum D Pereda MP Coso OA. Activation and induction of NUR77/NURR1 in corticotrophs by CRH/cAMP: involvement of calcium, protein kinase A, and MAPK pathways. Mol Endocrinol. (2002) 16:1638–51. 10.1210/mend.16.7.0863 12089357

[16] Kageyama K Hanada K Moriyama T Imaizumi T Satoh K Suda T. Differential regulation of CREB and ERK phosphorylation through corticotropin-releasing factor receptors type 1 and 2 in AtT-20 and A7r5 cells. Mol Cell Endocrinol. (2007) 263:90–102. 10.1016/j.mce.2006.08.011 17027144

[17] Traver S Marien M Martin E Hirsch EC Michel PP. The phenotypic differentiation of locus ceruleus noradrenergic neurons mediated by brain-derived neurotrophic factor is enhanced by corticotropin releasing factor through the activation of a cAMP-dependent signaling pathway. Mol Pharmacol. (2006) 70:30–40. 10.1124/mol.106.022715 16569708

[18] Pedersen WA Wan R Zhang P Mattson MP Urocortin, but not urocortin II, protects cultured hippocampal neurons from oxidative and excitotoxic cell death via corticotropin-releasing hormone receptor type I. J Neurosci. (2002) 22:404–12. 10.1523/JNEUROSCI.22-02-00404.2002 11784785 PMC6758668

[19] Brar BK Jonassen AK Egorina EM Chen A Negro A Perrin MH. Urocortin-II and urocortin-III are cardioprotective against ischemia reperfusion injury: an essential endogenous cardioprotective role for corticotropin releasing factor receptor type 2 in the murine heart. Endocrinology. (2004) 145:24–35; discussion 21-3. 10.1210/en.2003-0689 12970163

[20] Yuan Z McCauley R Chen-Scarabelli C Abounit K Stephanou A Barry SP. Activation of Src protein tyrosine kinase plays an essential role in urocortin-mediated cardioprotection. Mol Cell Endocrinol. (2010) 325:1–7. 10.1016/j.mce.2010.04.013 20416357

[21] Markovic D Punn A Lehnert H Grammatopoulos DK. Molecular determinants and feedback circuits regulating type 2 CRH receptor signal integration. Biochim Biophys Acta-Mol Cell Res. (2011) 1813:896–907. 10.1016/j.bbamcr.2011.02.005 21338628

[22] Dautzenberg FM Kilpatrick GJ Wille S Hauger RL. The ligand-selective domains of corticotropin-releasing factor type 1 and type 2 receptor reside in different extracellular domains: generation of chimeric receptors with a novel ligand-selective profile. J Neurochem. (1999) 73:821–9. 10.1046/j.1471-4159.1999.0730821.x 10428081

[23] Oakley RH Laporte SA Holt JA Caron MG Barak LS. Differential affinities of visual arrestin, beta arrestin1, and beta arrestin2 for G protein-coupled receptors delineate two major classes of receptors. J Biol Chem. (2000) 275:17201–10. 10.1074/jbc.M910348199 10748214

[24] Oakley RH Olivares-Reyes JA Hudson CC Flores-Vega F Dautzenberg FM Hauger RL. Carboxyl-terminal and intracellular loop sites for CRF1 receptor phosphorylation and β-arrestin-2 recruitment: a mechanism regulating stress and anxiety responses. Am J Physiol Regul Integr Comp Physiol. (2007) 293:R209–22. 10.1152/ajpregu.00099.2006 17363685 PMC3102763

[25] Koch WJ Hawes BE Inglese J Luttrell LM Lefkowitz RJ. Cellular expression of the carboxyl terminus of a G protein-coupled receptor kinase attenuates G beta gamma-mediated signaling. J Biol Chem. (1994) 269:6193–7. 8119963

[26] Servitja JM Marinissen MJ Sodhi A Bustelo XR Gutkind JS. Rac1 function is required for Src-induced transformation. Evidence of a role for Tiam1 and Vav2 in Rac activation by Src. J Biol Chem. (2003) 278:34339–46. 10.1074/jbc.M302960200 12810717

[27] Hauger RL Olivares-Reyes JA Braun S Catt KJ Dautzenberg FM. Mediation of corticotropin releasing factor type 1 receptor phosphorylation and desensitization by protein kinase C: a possible role in stress adaptation. J Pharmacol Exp Ther. (2003) 306:794–803. 10.1124/jpet.103.050088 12734388

[28] Shah BH Olivares-Reyes JA Catt KJ. The protein kinase C inhibitor Go6976 [12-(2-Cyanoethyl)-6,7,12,13-tetrahydro-13-methyl-5-oxo-5H-indolo(2,3-a)pyrrolo(3,4-c)-carbazole] potentiates agonist-induced mitogen-activated protein kinase activation through tyrosine phosphorylation of the epidermal growth factor receptor. Mol Pharmacol. (2005) 67:184. 10.1124/mol.104.003533 15465928

[29] Olivares-Reyes J Shah B Hernandez-Aranda J Garcia-Caballero A Farshori M Garcia-Sainz J Agonist-induced interactions between angiotensin AT(1) and epidermal growth factor receptors. Mol Pharmacol. (2005) 68:356–64. 10.1124/mol.104.010637 15905421

[30] Milan-Lobo L Gsandtner I Gaubitzer E Runzler D Buchmayer F Kohler G. Subtype-specific differences in corticotropin-releasing factor receptor complexes detected by fluorescence spectroscopy. Mol Pharmacol. (2009) 76:1196–210. 10.1124/mol.109.059139 19755522 PMC4503342

[31] Hawes BE van Biesen T Koch WJ Luttrell LM Lefkowitz RJ. Distinct pathways of Gi- and Gq-mediated mitogen-activated protein kinase activation. J Biol Chem. (1995) 270:17148–53. 10.1074/jbc.270.29.17148 7615510

[32] Luttrell LM Hawes BE van Biesen T Luttrell DK Lansing TJ Lefkowitz RJ. Role of c-Src tyrosine kinase in G protein-coupled receptor- and Gbetagamma subunit-mediated activation of mitogen-activated protein kinases. J Biol Chem. (1996) 271:19443–50. 10.1074/jbc.271.32.19443 8702633

[33] Rozengurt E. Mitogenic signaling pathways induced by G protein-coupled receptors. J Cell Physiol. (2007) 213:589–602. 10.1002/jcp.21246 17786953

[34] Luttrell LM. Composition and function of G protein-coupled receptor signalsomes controlling mitogen-activated protein kinase activity. J Mol Neurosci. (2005) 26:253–64. 10.1385/JMN:26:2-3:253 16012199

[35] Wee P Wang Z. Epidermal growth factor receptor cell proliferation signaling pathways. Cancers. (2017) 9:E52. 10.3390/cancers9050052 28513565 PMC5447962

[36] Wright JD Reuter CWM Weber MJ. Identification of sites on epidermal growth factor receptors which are phosphorylated by pp60src *in vitro*. Biochim Biophys Acta. (1996) 1312:85–93. 10.1016/0167-4889(96)00027-4 8672543

[37] Jorissen RN Walker F Pouliot N Garrett TP Ward CW Burgess AW. Epidermal growth factor receptor: mechanisms of activation and signalling. Exp Cell Res. (2003) 284:31–53. 10.1016/S0014-4827(02)00098-8 12648464

[38] Eguchi S Dempsey PJ Frank GD Motley ED Inagami T Activation of MAPKs by angiotensin II in vascular smooth muscle cells. Metalloprotease-dependent EGF receptor activation is required for activation of ERK and p38 MAPK but not for JNK. J Biol Chem. (2001) 276:7957–62. 10.1074/jbc.M008570200 11116149

[39] Prenzel N Zwick E Daub H Leserer M Abraham R Wallasch C. EGF receptor transactivation by G-protein-coupled receptors requires metalloproteinase cleavage of proHB-EGF. Nature. (1999) 402:884–8. 10.1038/47260 10622253

[40] Shah BH Baukal AJ Shah FB Catt KJ. Mechanisms of extracellularly regulated kinases 1/2 activation in adrenal glomerulosa cells by lysophosphatidic acid and epidermal growth factor. Mol Endocrinol. (2005) 19:2535–48. 10.1210/me.2005-0082 15928312

[41] Shah B Yesilkaya A Olivares-Reyes J Chen H Hunyady L Catt K. Differential pathways of angiotensin II-induced extracellularly regulated kinase 1/2 phosphorylation in specific cell types: role of heparin-binding epidermal growth factor. Mol Endocrinol. (2004) 18:2035–48. 10.1210/me.2003-0476 15143154

[42] Sun Y McGarrigle D Huang XY When a G protein-coupled receptor does not couple to a G protein. Mol Biosyst. (2007) 3:849–54. 10.1039/b706343a 18000562

[43] Luttrell DK Luttrell LM. Not so strange bedfellows: G-protein-coupled receptors and Src family kinases. Oncogene. (2004) 23:7969–78. 10.1038/sj.onc.1208162 15489914

[44] Dikic I Tokiwa G Lev S Courtneidge SA Schlessinger J. A role for Pyk2 and Src in linking G-protein-coupled receptors with MAP kinase activation. Nature. (1996) 383:547–50. 10.1038/383547a0 8849729

[45] Sato K Sato A Aoto M Fukami Y. c-Src phosphorylates epidermal growth factor receptor on tyrosine 845. Biochem Biophys Res Commun. (1995) 215:1078–87. 10.1006/bbrc.1995.2574 7488034

[46] Sato K. Cellular functions regulated by phosphorylation of EGFR on Tyr845. Int J Mol Sci. (2013) 14:10761–90. 10.3390/ijms140610761 23702846 PMC3709701

[47] Perkovska S Mejean C Ayoub MA Li J Hemery F Corbani M. V1b vasopressin receptor trafficking and signaling: role of arrestins, G proteins and Src kinase. Traffic. (2018) 19:58–82. 10.1111/tra.12535 29044966

[48] Shah BH Catt KJ. Calcium-independent activation of extracellularly regulated kinases 1 and 2 by angiotensin II in hepatic C9 cells: roles of protein kinase Cdelta, Src/proline-rich tyrosine kinase 2, and epidermal growth receptor trans-activation. Mol Pharmacol. (2002) 61:343–51. 10.1124/mol.61.2.343 11809859

[49] Vanhaesebroeck B Guillermet-Guibert J Graupera M Bilanges B. The emerging mechanisms of isoform-specific PI3K signalling. Nat Rev Mol Cell Biol. (2010) 11:329–41. 10.1038/nrm2882 20379207

[50] Hawes BE Luttrell LM van Biesen T Lefkowitz RJ. Phosphatidylinositol 3-kinase is an early intermediate in the G beta gamma-mediated mitogen-activated protein kinase signaling pathway. J Biol Chem. (1996) 271:12133–6. 10.1074/jbc.271.21.12133 8647803

[51] Bisotto S Fixman ED. Src-family tyrosine kinases, phosphoinositide 3-kinase and Gab1 regulate extracellular signal-regulated kinase 1 activation induced by the type A endothelin-1 G-protein-coupled receptor. Biochem J. (2001) 360:77–85. 10.1042/bj3600077 11695994 PMC1222204

[52] Laffargue M Raynal P Yart A Peres C Wetzker R Roche S. An epidermal growth factor receptor/Gab1 signaling pathway is required for activation of phosphoinositide 3-kinase by lysophosphatidic acid. J Biol Chem. (1999) 274:32835–41. 10.1074/jbc.274.46.32835 10551845

[53] Yart A Roche S Wetzker R Laffargue M Tonks N Mayeux P. A function for phosphoinositide 3-kinase beta lipid products in coupling beta gamma to Ras activation in response to lysophosphatidic acid. J Biol Chem. (2002) 277:21167–78. 10.1074/jbc.M110411200 11916960

[54] Liu L Xie Y Lou L PI3K is required for insulin-stimulated but not EGF-stimulated ERK1/2 activation. Eur J Cell Biol. (2006) 85:367–74. 10.1016/j.ejcb.2005.11.005 16406609

[55] Sampaio C Dance M Montagner A Edouard T Malet N Perret B. Signal strength dictates phosphoinositide 3-kinase contribution to Ras/extracellular signal-regulated kinase 1 and 2 activation via differential Gab1/Shp2 recruitment: consequences for resistance to epidermal growth factor receptor inhibition. Mol Cell Biol. (2008) 28:587–600. 10.1128/MCB.01318-07 18025104 PMC2223412

[56] Duckworth BC Cantley LC. Conditional inhibition of the mitogen-activated protein kinase cascade by wortmannin. Dependence on signal strength. J Biol Chem. (1997) 272:27665–70. 10.1074/jbc.272.44.27665 9346906

[57] New DC Wu K Kwok AW Wong YH. G protein-coupled receptor-induced Akt activity in cellular proliferation and apoptosis. FEBS J. (2007) 274:6025–36. 10.1111/j.1742-4658.2007.06116.x 17949438

[58] Arcaro A Aubert M Espinosa del Hierro ME Khanzada UK Angelidou S Tetley TD. Critical role for lipid raft-associated Src kinases in activation of PI3K-Akt signalling. Cell Signal. (2007) 19:1081–92. 10.1016/j.cellsig.2006.12.003 17275257

[59] Pleiman CM Hertz WM Cambier JC. Activation of phosphatidylinositol-3′ kinase by Src-family kinase SH3 binding to the p85 subunit. Science. (1994) 263:1609–12. 10.1126/science.8128248 8128248

[60] Strungs EG Luttrell LM. Arrestin-dependent activation of ERK and Src family kinases. Handb Exp Pharmacol. (2014) 219:225–57. 10.1007/978-3-642-41199-1_12 24292833

[61] Menard L Ferguson SS Zhang J Lin FT Lefkowitz RJ Caron MG. Synergistic regulation of beta2-adrenergic receptor sequestration: intracellular complement of beta-adrenergic receptor kinase and beta-arrestin determine kinetics of internalization. Mol Pharmacol. (1997) 51:800–8. 10.1124/mol.51.5.800 9145918

[62] Luttrell LM Ferguson SS Daaka Y Miller WE Maudsley S Della Rocca GJ. Beta-arrestin-dependent formation of beta2 adrenergic receptor-Src protein kinase complexes. Science. (1999) 283:655–61. 10.1126/science.283.5402.655 9924018

[63] Holmes KD Babwah AV Dale LB Poulter MO Ferguson SS. Differential regulation of corticotropin releasing factor 1alpha receptor endocytosis and trafficking by beta-arrestins and Rab GTPases. J Neurochem. (2006) 96:934–49. 10.1111/j.1471-4159.2005.03603.x 16412099

[64] Inda C Armando NG Dos Santos Claro PA Silberstein S. Endocrinology and the brain: corticotropin-releasing hormone signaling. Endocr Connect. (2017) 6:R99–120. 10.1530/EC-17-0111 28710078 PMC5551434

[65] Papadopoulou N Chen J Randeva HS Levine MA Hillhouse EW Grammatopoulos DK. Protein kinase A-induced negative regulation of the corticotropin-releasing hormone R1alpha receptor-extracellularly regulated kinase signal transduction pathway: the critical role of Ser301 for signaling switch and selectivity. Mol Endocrinol. (2004) 18:624–39. 10.1210/me.2003-0365 14657255

[66] Robichaux WG Cheng X. Intracellular cAMP sensor EPAC: physiology, pathophysiology, and therapeutics development. Physiol Rev. (2018) 98:919–1053. 10.1152/physrev.00025.2017 29537337 PMC6050347

[67] Van Kolen K Dautzenberg FM Verstraeten K Royaux I De Hoogt R Gutknecht E. Corticotropin releasing factor-induced ERK phosphorylation in AtT20 cells occurs via a cAMP-dependent mechanism requiring EPAC2. Neuropharmacology. (2010) 58:135–44. 10.1016/j.neuropharm.2009.06.022 19573542

[68] Ma YC Huang J Ali S Lowry W Huang XY. Src tyrosine kinase is a novel direct effector of G proteins. Cell. (2000) 102:635–46. 10.1016/S0092-8674(00)00086-6 11007482

[69] Gao Y Tang S Zhou S Ware JA. The thromboxane A2 receptor activates mitogen-activated protein kinase *via* protein kinase C-dependent Gi coupling and Src-dependent phosphorylation of the epidermal growth factor receptor. J Pharmacol Exp Ther. (2001) 296:426–33. 11160627

[70] Luttrell LM la Rocca GJ van Biesen T Luttrell DK Lefkowitz RJ Gbeta gamma subunits mediate Src-dependent phosphorylation of the epidermal growth factor receptor. A scaffold for G protein-coupled receptor-mediated Ras activation. J Biol Chem. (1997) 272:4637–44. 10.1074/jbc.272.7.4637 9020193

[71] Della Rocca GJ van Biesen T Daaka Y Luttrell DK Luttrell LM Lefkowitz RJ. Ras-dependent mitogen-activated protein kinase activation by G protein-coupled receptors: convergence of Gi- and Gq-mediated pathways on calcium/calmodulin, Pyk2, and Src kinase. J Biol Chem. (1997) 272:19125–32. 10.1074/jbc.272.31.19125 9235901

[72] Wells V Downward J Mallucci L Functional inhibition of PI3K by the [beta]GBP molecule suppresses Ras-MAPK signalling to block cell proliferation. Oncogene. (2007) 26:7709–14. 10.1038/sj.onc.1210580 17603562

[73] Kimura Y Takahashi K Totsune K Muramatsu Y Kaneko C Darnel AD. Expression of urocortin and corticotropin-releasing factor receptor subtypes in the human heart. J Clin Endocrinol Metab. (2002) 87:340–6. 10.1210/jcem.87.1.8160 11788672

[74] Takahashi K Totsune K Murakami O Saruta M Nakabayashi M Suzuki T. Expression of urocortin III/stresscopin in human heart and kidney. J Clin Endocrinol Metab. (2004) 89:1897–903. 10.1210/jc.2003-031663 15070962

[75] Ikeda K Tojo K Inada Y Takada Y Sakamoto M Lam M. Regulation of urocortin I and its related peptide urocortin II by inflammatory and oxidative stresses in HL-1 cardiomyocytes. J Mol Endocrinol. (2009) 42:479–89. 10.1677/JME-08-0151 19318426

[76] Ikeda K Tojo K Otsubo C Udagawa T Hosoya T Tajima N. Effects of urocortin II on neonatal rat cardiac myocytes and non-myocytes. Peptides. (2005) 26:2473–81. 10.1016/j.peptides.2005.05.021 16005543

[77] Chen-Scarabelli C Saravolatz L II McCaukey R Scarabelli G Di Rezze J Mohanty B. The cardioprotective effects of urocortin are mediated *via* activation of the Src tyrosine kinase-STAT3 pathway. JAK-STAT. (2013) 2:e24812. 10.4161/jkst.24812 24069562 PMC3772114

[78] Coste SC Kesterson RA Heldwein KA Stevens SL Heard AD Hollis JH. Abnormal adaptations to stress and impaired cardiovascular function in mice lacking corticotropin-releasing hormone receptor-2. Nat Genet. (2000) 24:403–9. 10.1038/74255 10742107

[79] Paneda C Winsky-Sommerer R Boutrel B de Lecea L. The corticotropin-releasing factor-hypocretin connection: implications in stress response and addiction. Drug News Perspect. (2005) 18:250–5. 10.1358/dnp.2005.18.4.908659 16034481

[80] Pelissier-Rota M Chartier NT Bonaz B Jacquier-Sarlin MR. A crosstalk between muscarinic and CRF2 receptors regulates cellular adhesion properties of human colon cancer cells. Biochimi Biophys Acta-Mol Cell Res. (2017) 1864:1246–59. 10.1016/j.bbamcr.2017.04.008 28432022

[81] Ducarouge B Pelissier-Rota M Laine M Cristina N Vachez Y Scoazec JY. CRF2 signaling is a novel regulator of cellular adhesion and migration in colorectal cancer cells. PLoS ONE. (2013) 8:e79335. 10.1371/journal.pone.0079335 24260200 PMC3832608

[82] Grisanti LA Guo S Tilley DG. Cardiac GPCR-mediated EGFR transactivation: impact and therapeutic implications. J Cardiovasc Pharmacol. (2017) 70:3–9. 10.1097/FJC.0000000000000462 28059858 PMC5516955

[83] Maudsley S Pierce KL Zamah AM Miller WE Ahn S Daaka Y The β2-adrenergic receptor mediates extracellular signal-regulated kinase activation *via* assembly of a multi-receptor complex with the epidermal growth factor receptor. J Biol Chem. (2000) 275:9572–80. 10.1074/jbc.275.13.9572 10734107

[84] Tilley DG Kim IM Patel PA Violin JD Rockman HA. beta-Arrestin mediates beta1-adrenergic receptor-epidermal growth factor receptor interaction and downstream signaling. J Biol Chem. (2009) 284:20375–86. 10.1074/jbc.M109.005793 19509284 PMC2740462

[85] Fan G-f Shumay E Malbon CC Wang H-y c-Src tyrosine kinase binds the β2-adrenergic receptor *via* phospho-Tyr-350, phosphorylates G-protein-linked receptor kinase 2, and mediates agonist-induced receptor desensitization. J Biol Chem. (2001) 276:13240–7. 10.1074/jbc.M011578200 11278940

[86] Laporte SA Scott MGH. β-arrestins: multitask scaffolds orchestrating the where and when in cell signalling. In: Scott MGH Laporte SA editors. Beta-Arrestins: Methods and Protocols. New York, NY: Springer New York (2019). p. 9–55. 10.1007/978-1-4939-9158-7_2 30919345

[87] Perry SJ Junger S Kohout TA Hoare SR Struthers RS Grigoriadis DE. Distinct conformations of the corticotropin releasing factor type 1 receptor adopted following agonist and antagonist binding are differentially regulated. J Biol Chem. (2005) 280:11560–8. 10.1074/jbc.M412914200 15653688

[88] Bonfiglio JJ Inda C Senin S Maccarrone G Refojo D Giacomini D. B-Raf and CRHR1 internalization mediate biphasic ERK1/2 activation by CRH in hippocampal HT22 Cells. Mol Endocrinol. (2013) 27:491–510. 10.1210/me.2012-1359 23371389 PMC5416933

[89] Dunn HA Chahal HS Caetano FA Holmes KD Yuan GY Parikh R. PSD-95 regulates CRFR1 localization, trafficking and β-arrestin2 recruitment. Cell Signal. (2016) 28:531–40. 10.1016/j.cellsig.2016.02.013 26898829

[90] Flood DG Finn JP Walton KM Dionne CA Contreras PC Miller MS. Immunolocalization of the mitogen-activated protein kinases p42MAPK and JNK1, and their regulatory kinases MEK1 and MEK4, in adult rat central nervous system. J Comp Neurol. (1998) 398:373–92. 10.1002/(SICI)1096-9861(19980831)398:3<373::AID-CNE6>3.0.CO;2-X 9714150

[91] Ross CA Wright GE Resh MD Pearson RC Snyder SH. Brain-specific src oncogene mRNA mapped in rat brain by *in situ* hybridization. Proc Natl Acad Sci USA. (1988) 85:9831–5. 10.1073/pnas.85.24.9831 3200860 PMC282875

[92] Sugrue MM Brugge JS Marshak DR Greengard P Gustafson EL. Immunocytochemical localization of the neuron-specific form of the c-src gene product, pp60c-src(+), in rat brain. J Neurosci. (1990) 10:2513–27. 10.1523/JNEUROSCI.10-08-02513.1990 1696980 PMC6570279

[93] Pawlak R Magarinos AM Melchor J McEwen B Strickland S. Tissue plasminogen activator in the amygdala is critical for stress-induced anxiety-like behavior. Nat Neurosci. (2003) 6:168–74. 10.1038/nn998 12524546

[94] Yang CH Huang CC Hsu KS. Behavioral stress modifies hippocampal synaptic plasticity through corticosterone-induced sustained extracellular signal-regulated kinase/mitogen-activated protein kinase activation. J Neurosci. (2004) 24:11029–34. 10.1523/JNEUROSCI.3968-04.2004 15590919 PMC6730281

[95] Refojo D Echenique C Muller MB Reul JM Deussing JM Wurst W. Corticotropin-releasing hormone activates ERK1/2 MAPK in specific brain areas. Proc Natl Acad Sci USA. (2005) 102:6183–8. 10.1073/pnas.0502070102 15833812 PMC1087957

[96] Krishnan V Han MH Mazei-Robison M Iniguez SD Ables JL Vialou V. AKT signaling within the ventral tegmental area regulates cellular and behavioral responses to stressful stimuli. Biol Psychiatry. (2008) 64:691–700. 10.1016/j.biopsych.2008.06.003 18639865 PMC2742561

[97] Dahlhoff M Siegmund A Golub Y Wolf E Holsboer F Wotjak CT AKT/GSK-3[beta]/[beta]-catenin signalling within hippocampus and amygdala reflects genetically determined differences in posttraumatic stress disorder like symptoms. Neuroscience. (2010) 169:1216–26. 10.1016/j.neuroscience.2010.05.066 20576499

