# Supplementary material for: CRF1 Receptor Signaling via the ERK1/2-MAP and Akt Kinase Cascades: Roles of Src, EGF Receptor, and PI3-Kinase Mechanisms
Source: Front Endocrinol (Lausanne). 2019 Dec 12;10:869. doi: 10.3389/fendo.2019.00869 (PMC6921279; doi:10.3389/fendo.2019.00869)
Supplement: Supplementary file 1 [file Data_Sheet_1.pdf]

SUPPLEMENTARY MATERIAL

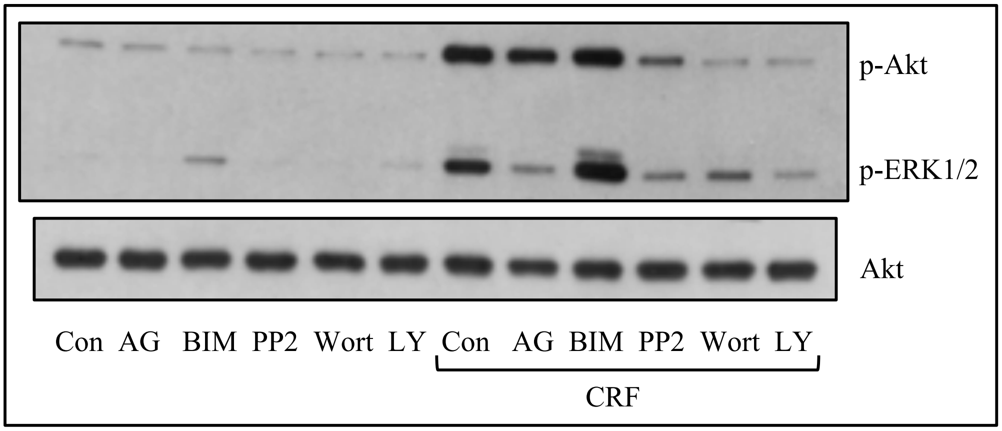

Supplementary Figure S1. ***Effect of protein kinase inhibitors under basal conditions and CRF-stimulated Akt and ERK1/2 phosphorylation.*** COS-7 cells expressing CRF$_1$Rs were treated with 100 nM AG1478 (AG), 1 µM BIM, 10 µM PP2, 100 nM wortmannin (Wort), or 10 µM LY294002 (LY) for 30 min or pretreated with the same inhibitors for 30 min before stimulation with 100 nM CRF for 5 min. Total cell lysates were separated by SDS-PAGE and analyzed by immunoblotting with anti-p-Akt Ser^{473} or anti-p-ERK1/2 Thr^{202}/Tyr^{204}, as described in *materials and methods*. A representative immunoblot is presented from three independent experiments. Western blots were also probed for total Akt, showing equal loading.

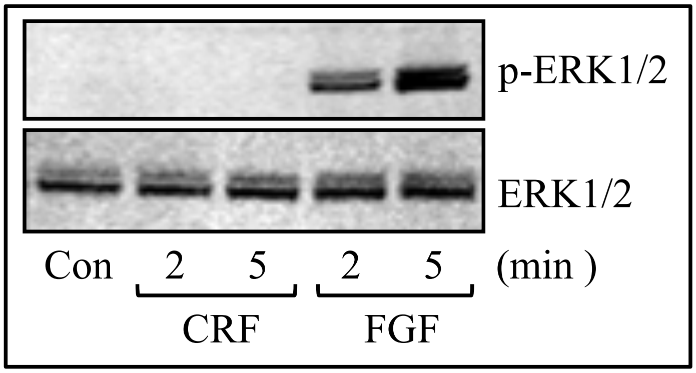

Supplementary Figure S2. ***CRF induces ERK1/2 phosphorylation in SK-N-MC neuroblastoma.*** CRF_1R expressing SK-N-MC neuroblastoma were stimulated with 100 nM CRF or 100 ng/ml fibroblast growth factor (FGF) for 2- or 5-min. Total cell lysates were separated by SDS-PAGE and analyzed by immunoblotting with anti-p-ERK1/2 Thr^{202}/Tyr^{204}, as described in *materials and methods*. A representative immunoblot is presented from three independent experiments. Western blots were also probed for total ERK1/2, showing equal loading.

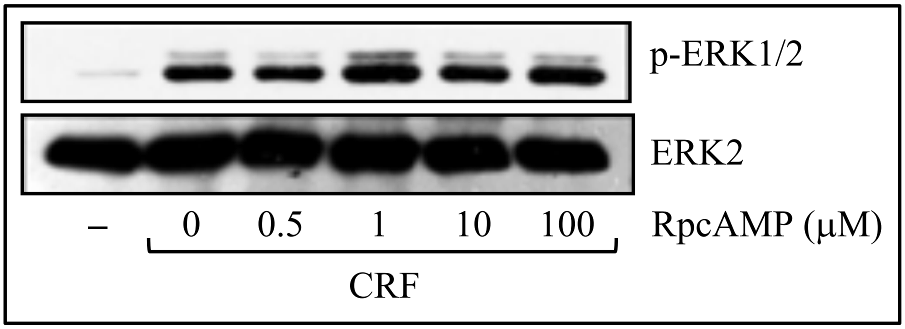

Supplementary Figure S3. ***Role of PKA in CRF-stimulated ERK1/2 phosphorylation.*** COS-7 cells expressing HA-CRF_1Rs were pretreated with the indicated concentrations of RpcAMP for 30 min before stimulation with 100 nM CRF for 5 min. Total cell lysates were separated by SDS-PAGE and analyzed by immunoblotting with anti-p-ERK1/2 Thr^{202}/Tyr^{204}, as described in *materials and methods*. A representative immunoblot is presented from three independent experiments. Western blots were also probed for total ERK2, showing equal loading.

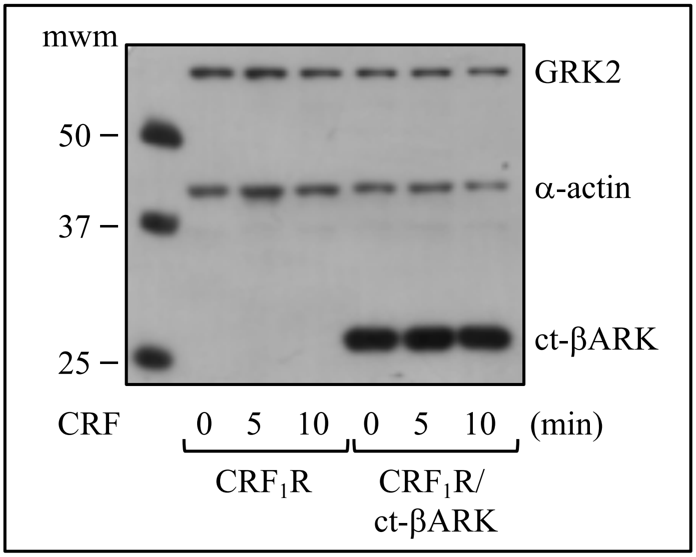

Supplementary Figure S4. ***ct-βARK overexpression in COS-7 cells.*** COS-7 cells co-transfected with a plasmid pRK5 encoding the carboxyl terminus of βARK that contains its βγ-binding domain (ct-βARK) or an empty control vector and the pcDNA3-HA-CRF_1R expression vector were stimulated with 100 nM CRF for the indicated times. Total cell lysates were separated by SDS-PAGE and analyzed by immunoblotting with anti-GRK2 or α-actin, as described in *materials and methods.* A representative immunoblot is presented from three independent experiments.

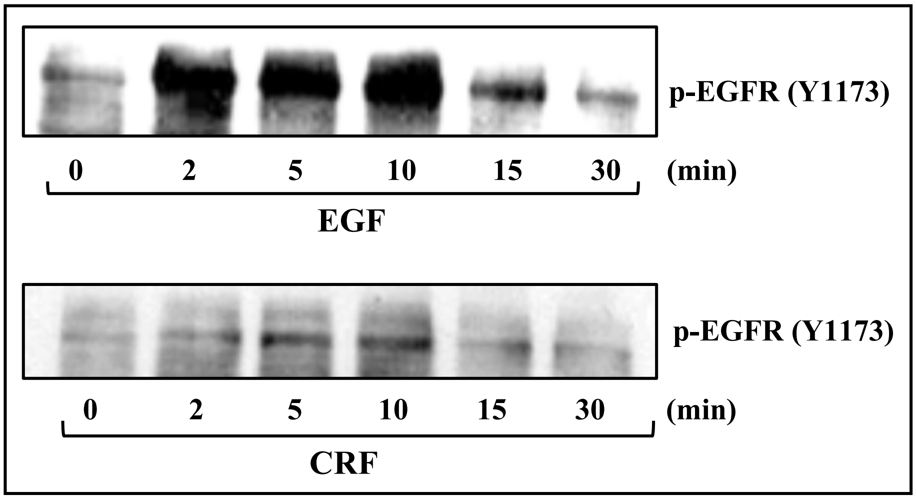

Supplementary Figure S5. ***Time-course dependent effect of EGF and CRF on EGFR activation.*** COS-7 cells expressing HA-CRF_1Rs were stimulated with 10 ng/ml EGF or 100 nM CRF for the indicated times. Total cell lysates were separated by SDS-PAGE and analyzed by immunoblotting with anti-p-EGF Tyr^{1173}, as described in *materials and methods*. The blot is representative of three independent experiments.

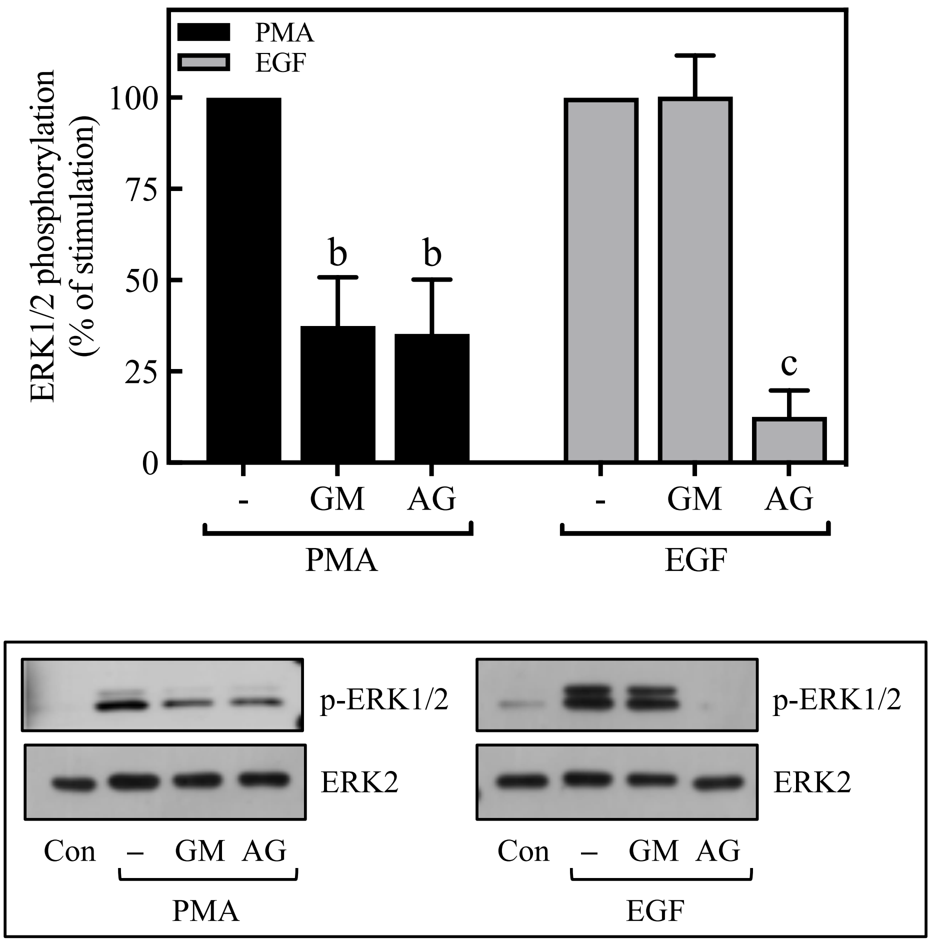

Supplementary Figure S6. ***Effect of the MMP and EGFR inhibitors on PMA- and EGF-stimulated ERK1/2 phosphorylation.*** COS-7 cells expressing HA-CRF_1Rs were pretreated with 10 μM GM6001 or 100 nM AG1478 for 30 min before stimulation with 100 nM PMA (15 min) or 10 ng/ml EGF (10 min). Total cell lysates were separated by SDS-PAGE and analyzed by immunoblotting with anti-p-ERK1/2 Thr^{202}/Tyr^{204}, as described in *materials and methods*. ERK1/2 phosphorylation was quantitated by densitometry, and mean values were plotted from three independent experiments. Vertical lines represent the S.E.M. Representative immunoblots are presented. Western blots were also probed for total ERK, showing equal loading. $^{b}p < 0.01$ *vs* PMA (-); $^{c}p<0.001$ *vs* EGF (-).

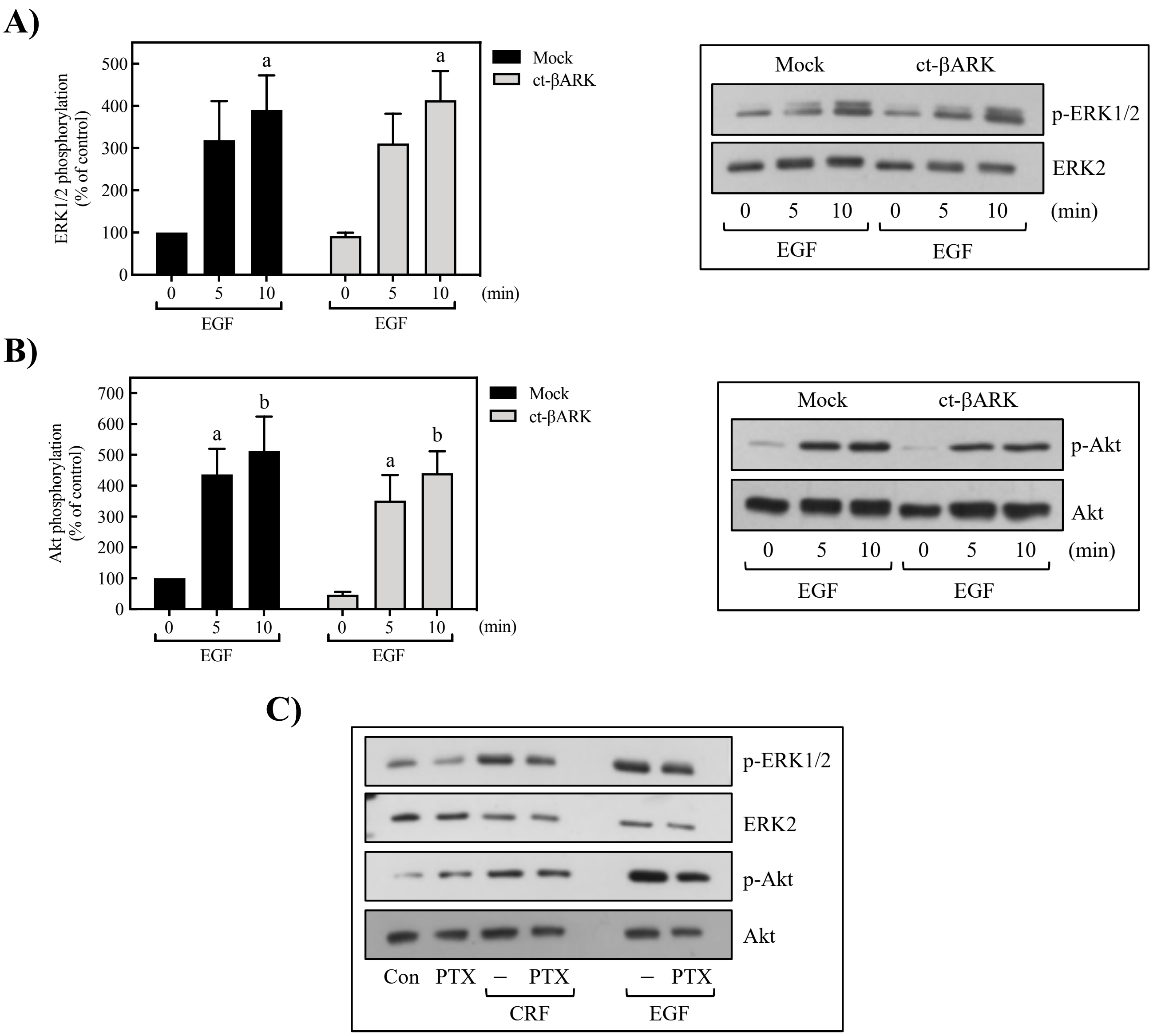

Supplementary Figure S7. ***Effect of PTX and ct-βARK on EGF-stimulated ERK1/2 and Akt phosphorylation.*** COS-7 cells transfected with an empty control vector (Mock) or a plasmid pRK5 encoding the carboxyl terminus of βARK that contains its βγ-binding domain (ct-βARK) were stimulated with 10 ng/ml EGF for 5 or 10 min**. (C)** COS-7 cells expressing HA- CRF_1Rs were pretreated with 100 ng/ml PTX for 15 h before stimulation with 100 nM CRF for 5 min or 10 ng/ml EGF for 5 min. Total cell lysates were separated by SDS-PAGE and analyzed by immunoblotting with anti-p-ERK1/2 Thr^{202}/Tyr^{204} **(A and C)** or anti-p-Akt Ser^{473} **(B and C)**, as described in *materials and methods*. ERK1/2 and Akt phosphorylation **(A and B, respectively)** were quantitated by densitometry, and mean values were plotted from three independent experiments. Vertical lines represent the S.E.M. Western blots were also probed for total ERK and Akt showing equal loading. **(A)** $^{a}p < 0.05$ *vs* 0 min (Mock); $^{a}p < 0.05$ *vs* 0 min (ct-βARK). **(B)** $^{a}p < 0.05$ *vs* 0 min, $^{b}p<0.01$ *vs* 0 min (Mock); $^{a}p < 0.05$ *vs* 0 min, $^{b}p<0.01$ *vs* 0 min (ct-βARK).
